# Proteomic and Bioinformatic Investigations of Heat-Treated *Anisakis simplex* Third-Stage Larvae

**DOI:** 10.3390/biom10071066

**Published:** 2020-07-16

**Authors:** Maciej Kochanowski, Mirosław Różycki, Joanna Dąbrowska, Aneta Bełcik, Jacek Karamon, Jacek Sroka, Tomasz Cencek

**Affiliations:** Department of Parasitology and Invasive Diseases, National Veterinary Research Institute, Partyzantów Avenue 57, 24-100 Puławy, Poland; mrozycki@piwet.pulawy.pl (M.R.); joanna.dabrowska@piwet.pulawy.pl (J.D.); aneta.belcik@piwet.pulawy.pl (A.B.); j.karamon@piwet.pulawy.pl (J.K.); jacek.sroka@piwet.pulawy.pl (J.S.); tcencek@piwet.pulawy.pl (T.C.)

**Keywords:** *Anisakis simplex*, foodborne parasite, allergen, potential allergen, hidden allergen, mass spectrometry, bioinformatics, proteome, autoclaving

## Abstract

*Anisakis simplex* third-stage larvae are the main source of hidden allergens in marine fish products. Some *Anisakis* allergens are thermostable and, even highly processed, could cause hypersensitivity reactions. However, *Anisakis* proteome has not been studied under autoclaving conditions of 121 °C for 60 min, which is an important process in the food industry. The aim of the study was the identification and characterization of allergens, potential allergens, and other proteins of heat-treated *A. simplex* larvae. Liquid chromatography-tandem mass spectrometry (LC-MS/MS) was used to identify 470 proteins, including allergens—Ani s 1, Ani s 2, Ani s 3, Ani s 4, Ani s 5—and 13 potential allergens that were mainly homologs of *Anisakis* spp., *Ascaris* spp., and Acari allergens. Ani s 2, Ani s 3, Ani s 5, and three possible allergens were found among the top 25 most abundant proteins. The computational analysis allowed us to detect allergen epitopes, assign protein families, and domains as well as to annotate the localization of proteins. The predicted 3D models of proteins revealed similarities between potential allergens and homologous allergens. Despite the partial degradation of heated *A. simplex* antigens, their immunoreactivity with anti-*A. simplex* IgG antibodies was confirmed using a Western blot. In conclusion, identified epitopes of allergenic peptides highlighted that the occurrence of *Anisakis* proteins in thermally processed fish products could be a potential allergic hazard. Further studies are necessary to confirm the IgE immunoreactivity and thermostability of identified proteins.

## 1. Introduction

Foodborne parasites are one of the most important causative agents of human infectious diseases, especially in less developed countries [1,2]. The climate changes, new feeding habits, and globalization of food supply chains may increase the worldwide incidence of some foodborne diseases [3,4]. Unfortunately, foodborne parasites remain neglected compared with bacterial and viral pathogens [3]. Therefore, studies in this field, like epidemiological surveys [5,6], development of novel diagnostic tools [7,8,9], drug discovery [10], or investigation of pathogenicity [11], are particularly valuable.

*Anisakis* spp. is one of the most important fish-borne parasites [2]. Live third-stage larvae (L3) of *Anisakis simplex* consumed with fish or seafood dishes can cause a human disease called anisakiasis. Over 20,000 cases of anisakiasis had been reported worldwide before 2010 [12]. Bao et al. [13] estimated that the total number of worldwide anisakidosis (almost all anisakiasis) cases up to December 2017 might be over 76,000. According to a report of the Orphanet (the portal of rare diseases and orphan drugs; January 2020), the worldwide incidence of anisakiasis is 0.32/100,000 [14,15]. However, due to very nonspecific symptoms of the disease, the number of detected cases is considered to be highly underestimated [16]. These assumptions confirm the results of the last study in which the number of anisakiasis cases requiring medical attention in Spain has been estimated at around 8000 per year [17]. 

*A. simplex* L3 larvae are also a known source of allergens, and 14 of them (Ani s 1–Anis 14) are officially recognized by the World Health Organization and International Union of Immunological Societies (WHO/IUIS) Allergen Nomenclature Sub-committee. The list of *Anisakis* allergens seems incomplete as new isoforms [18] and new potential allergens [19,20,21,22,23] are still being identified. *A. simplex* allergens are considered to be the most common hidden allergens in marine fish products [24]. These allergens may cause hypersensitivity reactions in sensitized humans in the form of urticaria, angioedema, asthma, rhinitis, conjunctivitis, gingivostomatitis, contact dermatitis, and even severe anaphylaxis [25,26]. Inhalation and contact allergy to *A. simplex* are mostly linked with occupational diseases of food industry employees, cooks, and restaurant workers [27,28,29,30]. Non-occupational airborne-induced anaphylaxis after exposure to *Anisakis* allergens has been also reported [31]. The allergic response caused by *Anisakis* could also be associated with nephrotic syndrome and rheumatic manifestations [32,33].

The prevalence of *Anisakis* hypersensitivity in symptomatic patients varies widely depending on geographical area, characteristics of the population studied, diagnostic criteria, and laboratory assays [30]. Researchers considered that, in endemic countries, the number of highly sensitized humans in the general population could be approximately 7% [34].

As several *A. simplex* allergens are known to be resistant to high and low temperatures or pepsin digestion, allergens of dead larvae may cause hypersensitivity reactions in sensitized humans [35]. Cases of *Anisakis*-related hypersensitivity reactions have been noted even after the ingestion of canned food [26] or meat of chicken fed with fishmeal containing *Anisakis* allergens [36]; however, *Anisakis* allergies after the consumption of processed food have not been well investigated. Similarly, the *A. simplex* allergome and proteome subjected to autoclaving have not been tested so far. This process is one of the most important food preservation methods in the industry and is widely used in the production of canned fish products. 

The autoclaving of proteins may result in a reduction of allergenicity by the disruption of IgE epitopes but, overall, it cannot be completely abolished [37]. Furthermore, thermal processing may generate new epitopes in the proteins, leading to neoallergen formation, which could cause an enhancement of allergenicity [38,39]. Therefore, the examination of the heat-treated allergens and possible allergens is important to determine the potential risk of hypersensitivity reactions for sensitized consumers of processed food. Mass spectrometry proteomics approaches seem to be a useful tool for this purpose as they allow for the high-throughput identification of proteins and have been successfully applied to many studies for the analysis of allergens in food [40,41].

In this context, the goal of our study was proteomic and computational investigations of heat-treated *A. simplex* L3 larvae. In addition to the mass spectrometry identification of proteins, we performed an investigation of the influence of high temperature on the antigenic profiles of *A. simplex* and the immunoreactivity of *Anisakis* antigen with IgG antibodies. Subsequently, the identified proteins were processed using a bioinformatics approach to characterize proteins with a particular focus on the allergenic peptides and their epitopes.

## 2. Materials and Methods 

### 2.1. Ethics Statement

Rabbits purchased from the Center for Experimental Medicine (Katowice, Poland) were housed under standard conditions, and experiments were conducted under the approval of the Local Ethical Commission for Animal Experimentation (license no: 66/2012).

### 2.2. A. simplex L3 Larvae Collection and Identification

Anisakis spp. L3 larvae were collected from marine fishes, as described previously [42]. The larvae were purified by washing with sterile 0.01 M phosphate-buffered saline solution (PBS, pH 7.4; Sigma, St. Louis, MO, USA). Then, the identification of species of Anisakis spp. nematodes was performed using PCR-restriction fragment length polymorphism (PCR-RFLP) [43].

### 2.3. Protein Extraction from A. simplex L3 Larvae

Three different antigens of *A. simplex* were obtained:

- the procedure for native crude (CR) antigens of *A. simplex* was performed, as previously reported [42];
- the procedure for heat-treated CR antigens of *A. simplex* was performed by the heating of native CR antigen in a thermomixer at 100 °C for 60 min;
- the procedure for heat-sterilized CR antigens of *A. simplex* was performed by the autoclaving of CR antigens at 121 °C for 60 min.

Protein concentration was determined by measuring the absorbance at 280 nm using an ultraviolet-visible (UV-vis) spectrophotometer (Implen, München, Germany) and adjusted to 1 mg/mL. Protein extracts were kept at –80 °C for further analysis.

### 2.4. Generation of Rabbit Anti-A. simplex Antiserum

Rabbits were immunized by intramuscular injection of 2 mg of A. simplex native CR antigen mixed with Freund’s complete adjuvant (Sigma, St. Louis, MO, USA). Immunization was performed according to our previously described protocol [42]. Preimmune serum was taken before immunization and used as a negative control. Sera were stored at –80 °C until use.

### 2.5. Sodium Dodecyl Sulfate-Polyacrylamide Gel Electrophoresis (SDS–PAGE) and IgG Western Blot (WB)

SDS-PAGE analysis of A. simplex antigens and WB reaction of antigens with rabbit anti-A. simplex serum was generally performed, as described previously [23], using 4-chloro-1-naphthol (Sigma, St. Louis, MO, USA) as a substrate for horseradish peroxidase (HRP)-conjugated goat anti-rabbit IgG antibodies (Sigma, St. Louis, MO, USA).

The molecular weight of the SDS-PAGE and WB bands were estimated using Bio-1D software (ver. 15.07; Vilber Lourmat, Marne-la-Vallée, France). ImageJ software (ver. 1.53a; National Institute of Health, Bethesda, MD, USA, *http://rsb.info.nih.gov/ij/*) was used for semi-quantitative density measurements of the SDS-PAGE and WB profile based on the integrated density (IntDen) calculation (the product of area and mean gray value) [44].

### 2.6. Sample Processing and Liquid Chromatography-Tandem Mass Spectrometry (LC-MS/MS) Analysis

The autoclaved A. simplex antigens (three independent biological replicates) were subjected to the standard procedure of tryptic digestion during which proteins were reduced with 0.5 M (5 mM f.c.) tris (2-carboxyethyl) phosphine (TCEP; Sigma, St. Louis, MO, USA) for 1 h at 60 °C, and the cysteine residues were subsequently alkylated with 200 mM (10 mM f.c.) methyl methanethiosulfonate (MMTS; Sigma, St. Louis, MO, USA) for 10 min at room temperature and finally cleaved overnight with 10 µL of 0.1 µg/µL trypsin (Promega, Madison, WI, USA) at 37 °C. The resulting peptide mixtures were loaded in equal volumes of 20 µL to a reversed-phase RP-18 pre-column (Waters, Milford, MA, USA) using 0.1% formic acid (FA; Sigma, St. Louis, MO, USA) in water as a mobile phase and then transferred to nano-high-performance liquid chromatography (nano-HPLC) RP-18 column (internal diameter 75 µm; Waters, Milford, MA, USA) using linear acetonitrile (ACN; Sigma, St. Louis, MO, USA) gradient 0–35% over 160 min in the presence of 0.1% FA at a flow rate of 250 nL/min. The nano-HPLC column outlet was coupled directly to the ion source of the Q Exactive mass spectrometer (Thermo Electron Corp., San Jose, CA, USA) working in the regime of data-dependent MS to MS/MS switch with higher-energy collisional dissociation (HCD) type peptide fragmentation. A blank run ensuring the absence of cross-contamination from previous samples preceded each analysis.

Mass spectrometric data were preprocessed with Mascot Distiller software (ver. 2.6; Matrix Science, London, UK; http://www.matrixscience.com/distiller.html) and analyzed with the Mascot search engine server (ver. 2.5; Matrix Science, London, UK; http://www.matrixscience.com/server.html) against the A. simplex reference proteome (20,786 sequences; proteome ID: UP000036680) obtained from the Universal Protein Resource (UniProt, http://www.uniprot.org/). To reduce mass errors, the peptide and fragment mass tolerance settings were established separately for individual LC-MS/MS runs after a measured mass recalibration, resulting in values of 5 ppm for the parent and 0.01 Da for the fragment ions in higher-energy collisional dissociation (HCD) MS/MS mode. Peptide sequences were searched using trypsin specificity, allowing one missed cleavage; ion type was set as monoisotopic, and protein mass as unrestricted. Beta-methylthiolation of cysteine was used as a fixed modification, whereas oxidation of methionine was set as a variable modification. A score threshold for all samples was set for 50 to match the highest score threshold computed by the Mascot software. Only proteins identified in all three biological replicates were accepted. Quantification of protein abundance was performed using the exponentially modified protein abundance index (emPAI) provided by Mascot. 

The mass spectrometry proteomics data were deposited to the ProteomeXchange Consortium via the PRIDE [45] partner repository with the dataset identifier PXD018059 and 10.6019/PXD018059.

### 2.7. Bioinformatic Analysis

The functional annotation of the identified proteins, including gene ontology (GO) and InterPro analyses, was performed using OmicsBox software (ver. 1.2.4; BioBam Bioinformatics SL, Valencia, Spain, https://www.biobam.com/omicsbox/) based on the Blast2GO annotation methodology [46]. Annotations were run with the default settings, as we previously described [23], and a list of annotations was filtered using nematode taxonomy to improve the prediction accuracy.

Experimentally verified epitopes in the peptides of Anisakis allergens were searched in the Immune Epitope Database (IEDB; last updated on June 14, 2020; https://www.iedb.org/). Allergens in which epitopes were not found in IEDB were subjected to in silico prediction for the detection of potential epitopes in the peptides of these allergens. Bioinformatic detection of potential epitopes was performed using DNASTAR Protean 3D software (ver. 17.0.2.1; DNASTAR, Madison, WI, USA). B-cell epitopes were predicted by applying a confidence threshold of 0.7. Possible epitopes of major histocompatibility complex class II (MHC II) molecules were detected using the default settings. The mapping of potential T-cell epitopes was performed by combining AMPHI and Rothbard-Taylor methods using the default settings.

The identified proteins were evaluated for putative allergenicity by searching against The Food Allergy Research and Resource Program (FARRP) AllergenOnline.org database (ver. 20; 2,171 sequences; February 10, 2020; http://www.allergenonline.com/) using full-length FASTA alignment (e-value cut-off: 1e-05; 70% identity match).

The ExPASy Compute pI/Mw tool (https://web.expasy.org/compute_pi/) was applied for the calculation of the theoretical isoelectric point (pI) and molecular weight (Mw) of detected proteins.

Unknown 3D structures of allergens were predicted by homology modeling using the Phyre2 server (http://www.sbg.bio.ic.ac.uk/phyre2/) in intensive mode (multi-template + ab initio) [47]. Known 3D structures of allergens were derived from RCSB Protein Data Bank (RCSB PDB; https://www.rcsb.org/). Visualization of the 3D structures of allergens and structural alignment of allergen models were performed using the PyMOL Molecular Graphics System (ver. 2.0; Schrödinger, LLC, New York, USA).

## 3. Results

### 3.1. Comparative SDS-PAGE and IgG-WB Analyses of A. simplex Antigens

**Figure 1:**
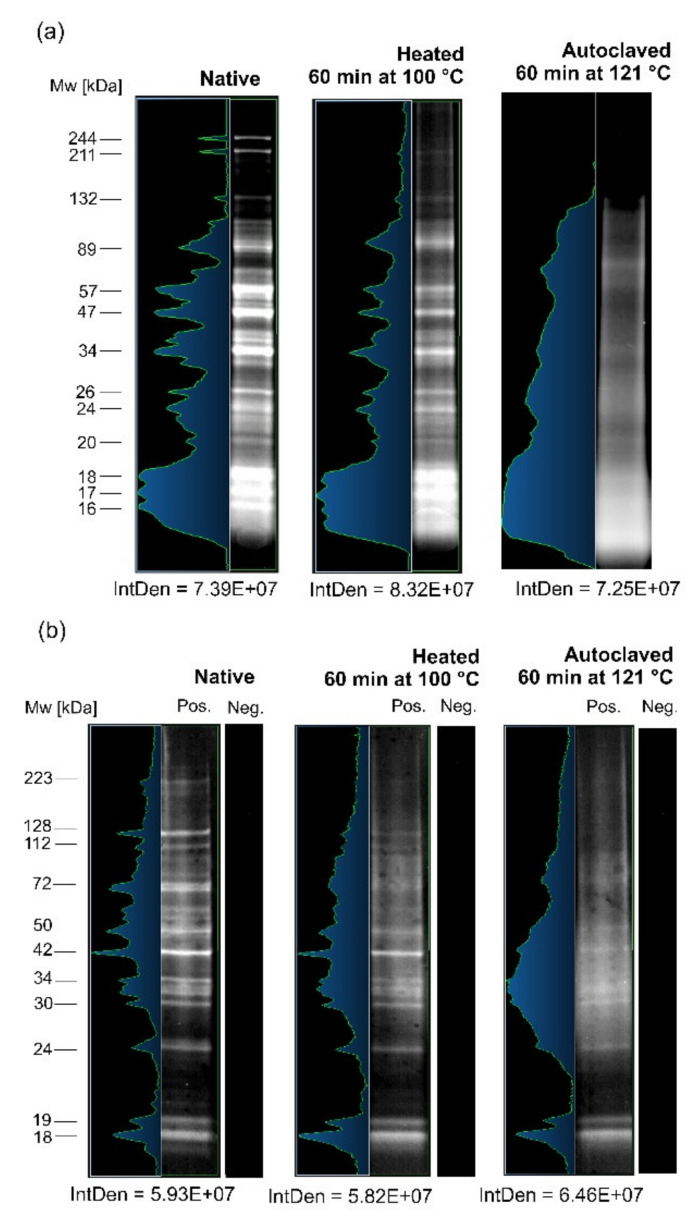
Colloidal Coomassie-stained SDS-PAGE analysis (color inversion mode) of the three following crude (CR) antigens of *A. simplex*: native, heated for 60 min at 100 °C, and autoclaved for 60 min at 121 °C (**a**). Western blot analysis of anti-*A. simplex* rabbit IgG antibodies reactivity against following CR antigens of *A. simplex*: native, heated 60 min at 100 °C, and autoclaved for 60 min at 121 °C (**b**). Pos.—membrane incubated with hyperimmune serum from a rabbit immunized with *A. simplex* CR antigen; Neg.—membrane incubated with rabbit preimmune serum. Molecular weight (Mw) estimations are presented in kilodaltons (kDa), as performed by Bio-1D software. The integrated density (IntDen) was calculated by ImageJ software.

SDS-PAGE and WB analyses were performed to investigate the influence of high temperature on the Anisakis antigen. Figure 1a shows the SDS-PAGE multiband profiles of native and heat-treated CR antigens of A. simplex. The bands’ profile of antigen heated for 60 min at 100 °C was similar to the native antigen, and just a few high molecular mass bands (132-244 kDa) that were present in the native antigen were not visible in the heated antigen. The intensity of bands was slightly lower compared to the native antigen. The SDS-PAGE profile of antigen autoclaved at 121 °C for 60 min was characterized by diffused band patterns with high background and reduced number of bands compared to the native or heated (at 100 °C) antigens. However, the bands were visible at the following molecular weights: 16‒18, 20, 24‒26, 34, and about 60 kDa. The IntDen values of all three SDS-PAGE profiles were very similar and ranged from 7.25E+07 to 8.32E+07.

The WB profiles of *Anisakis* antigens are presented in Figure 1b. The profiles of both heated antigens were generally consistent with the native antigen. Similarly to the SDS-PAGE profile, the number and intensity of bands were reduced, and a background appeared. The background in the WB profile of autoclaved antigen was slightly higher than in heated at 100 °C. The IntDen values were very similar for the WB profiles, and they were in the range from 5.82E+07 to 6.46E+07.

The background in the SDS and WB profiles of heated antigens was higher than in native antigen, probably due to degradation. However, the band pattern confirmed that degradation was only partial. Furthermore, the epitopes of degraded proteins probably were not damaged.

### 3.2. Identification and Characterization of A. simplex Proteins 

A total of 470 proteins were detected in all three biological replicates of shotgun LC-MS/MS analysis. The identification was performed with high confidence as the peptides false discovery rate (FDR) calculated by Mascot in all cases was 0.99%. A detailed list of all identified proteins with UniProt IDs, protein names, gene names, and OmicsBox annotations is presented in Supplemental File S1. 

**Figure 2:**
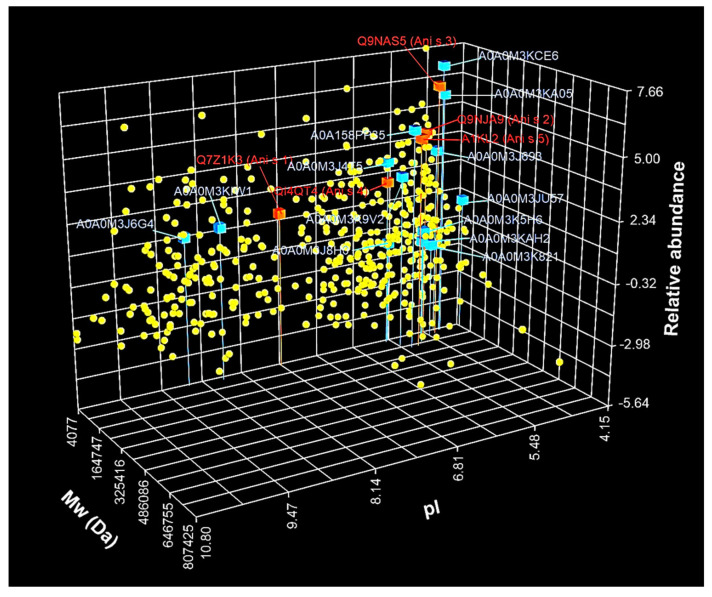
The three-dimensional scatter plot analysis of *A. simplex* proteins (*n* = 470) identified using LC-MS/MS. The proteins were distributed based on molecular weight (Mw), isoelectric point (pI), and the relative abundance of proteins. The Mw and pI values were calculated using the ExPASy Compute pI/Mw tool, while the relative abundance was estimated based on the average exponentially modified protein abundance index (emPAI) values (mean of three biological replicates) calculated by Mascot. The relative abundance is shown in log2 scale. Red cubes are allergens (*n* = 5), and cyan cubes are potential allergens (*n* = 13). Cubes (allergens and potential allergens) are signed with UniProt ID and have a vertical line for better pI and Mw visualization. Yellow spheres are the other identified proteins. A scatter plot was constructed using Teraplot software (ver. 1.4.06; Kylebank Software Ltd., Ayr, UK).

Detected proteins were displayed on the 3D scatter plot (Figure 2) based on theoretical Mw, theoretical pI, and estimated relative protein abundance. The molecular weights of all proteins ranged from 4077 to 807,425 Da. An uncharacterized protein (UniProt ID: A0A0M3JJQ2) had the lowest Mw from all detected proteins, while twitchin (UniProt ID: A0A158PN23) had the highest Mw. The majority of proteins (n = 396) were in the range of Mw from about 10 kDa to 94.19 kDa. In the case of the estimated pI values, proteins were in the range of 4.15-10.8. The lowest pI value was calculated for troponin-like protein (UniProt ID: A0A0M3JU57), whereas the highest value was estimated for 60S ribosomal protein L8 (UniProt ID: A0A0M3K2K1). The pI values for the majority of the proteins were in one of the two following ranges: 4.15-7.19 (n = 320) and 8-9.59 (n = 101). 

The protein abundance was estimated using emPAI, which provided an approximate protein relative quantification based on the number of observed peptides divided by the number of observable peptides. The estimated emPAI values of proteins by Mascot were in the range 0.02-201.76. Among all identified proteins, the lowest emPAI values were calculated for collagen alpha-1(IV) chain (emPAI = 0.02; UniProt ID: A0A158PPJ2), uncharacterized protein (emPAI = 0.03; UniProt ID: A0A158PP53), calcium-transporting ATPase (emPAI = 0.03; UniProt ID: A0A0M3JTY4), and uncharacterized protein (emPAI = 0.03; UniProt ID: A0A0M3K3G9). While the most abundant were the following proteins: myosin essential light chain (emPAI = 201.76; UniProt ID: A0A0M3K6N3), tropomyosin (emPAI = 109.71; UniProt ID: A0A0M3KCE6), and DUF4440 domain-containing protein (emPAI = 81.05; UniProt ID: A0A0M3J349).

Table 1 shows the top 25 most abundant proteins, with emPAI values in the range of 13.78‒201.76. The Mw and pI values of the majority of highly abundant proteins were in the ranges 10-29.9 kDa (n = 21) and 4.5‒6.39 (n = 20), respectively. The distribution of log2-transformed values of the emPAI of all proteins is illustrated in Figure 2. The average emPAI values were log2-transformed to normalize the data. As shown in Figure 2, most of the log2-transformed emPAI values (n = 413) were in the range from ‒4.2 to 2.99.

**Figure 3:**
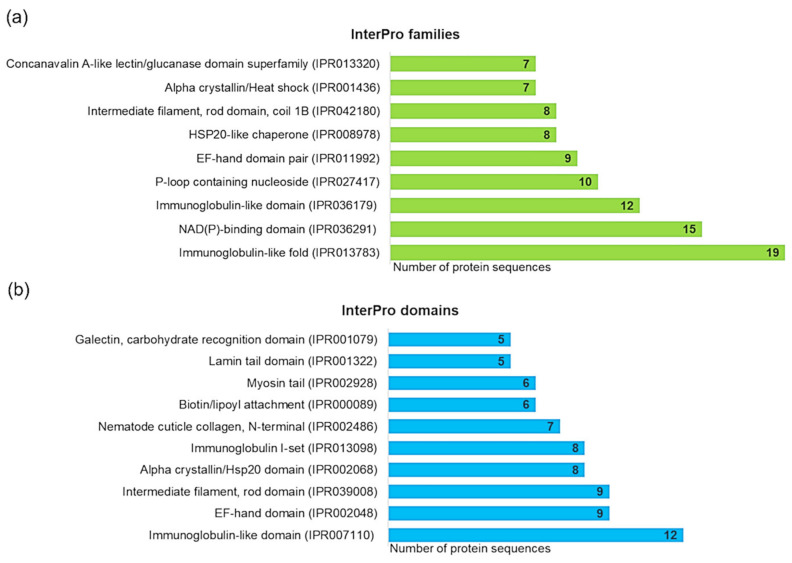
Results of the InterPro analysis of all detected proteins performed using OmicsBox software. Most abundant InterPro families (**a**); most abundant InterPro domains (**b**).

InterPro analysis was performed to characterize and classify detected proteins of A. simplex. Three hundred twenty different protein families and 203 domains were detected based on sequence analysis of all 470 proteins, and the most highly represented of them are displayed in Figure 3. A complete list of InterPro matches is presented in Supplemental File S1. The most abundant InterPro families (Figure 3a) were immunoglobulin-like fold (19 sequences), followed by the NAD(P)-binding domain superfamily (15 sequences), immunoglobulin-like domain superfamily (12 sequences), P-loop containing nucleoside triphosphate hydrolase (10 sequences), and EF-hand domain pair (9 sequences). Most of the identified protein families (n = 220) were represented only by one sequence. As shown in Figure 3b, the most abundant were the following InterPro domains: immunoglobulin-like domain (12 sequences), EF-hand domain (9 sequences), intermediate filament, rod domain (9 sequences), alpha-crystallin/Hsp20 domain (8 sequences), and intermediate filament rod domain coil 1B (8 sequences). One hundred thirty-six domains had only one assigned sequence.

InterPro analysis of the most abundant 25 proteins allowed the identification of 10 families and 9 domains (see Table 1). Among them, slightly more often (two matches for each) were reported the following proteins families: alpha-crystallin/heat shock protein, EF-hand domain pair, HSP20-like chaperone, intermediate filament rod domain coil 1B, and tropomyosin. Whereas the most abundant InterPro domain was the intermediate filament rod domain (three matches), followed by the alpha-crystallin/Hsp20 domain, domain of unknown function DUF148, EF-hand domain, and myosin tail (each of the two matches).

**Figure 4:**
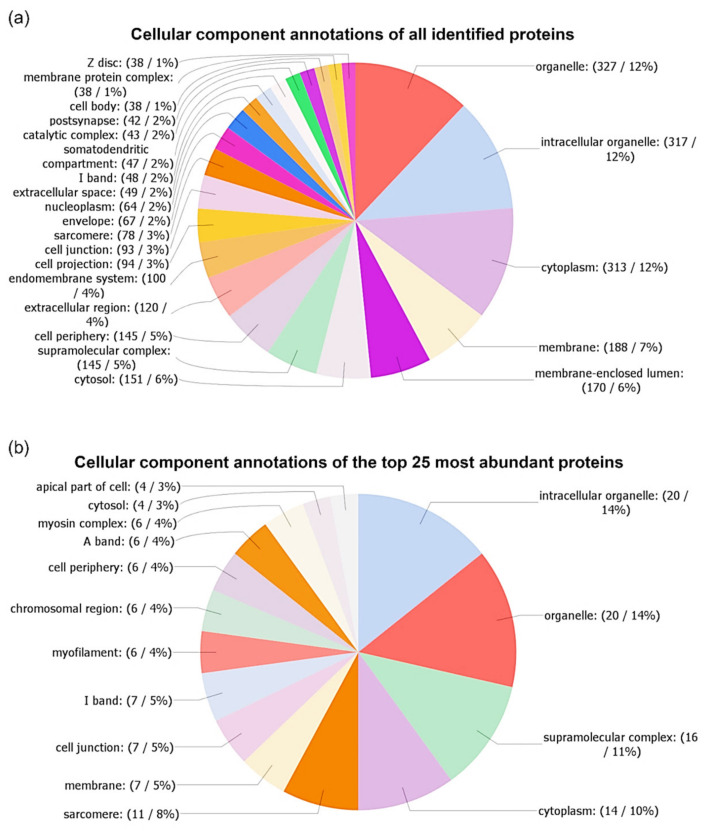
Gene ontology (GO) analysis of cellular components of all identified proteins (**a**), and the top 25 most abundant proteins (**b**). The number of annotations was limited for better readability in the following manner: a minimum of 38 matching sequences in GO analysis of all proteins, and a minimum of 4 matching sequences in the case of the top 25 most abundant proteins. Annotations were performed using OmicsBox software.

Gene ontology cellular component annotation was used to predict the localization of the detected proteins. Figure 4a shows the distribution of the cellular component annotations of all proteins. The most highly abundant cellular component annotations were the following: organelle (12%; GO:0043226), intracellular organelle (12%; GO:0043229), and cytoplasm (12%; GO:0005737). Less abundant were the following GO terms: membrane (7%; GO:0016020), membrane-enclosed lumen (6%; GO:0031974), cytosol (6%; GO:0005829), supramolecular complex (5%; GO:0099080), cell periphery (5%; GO:0071944), extracellular region (4%; GO:0005576), and endomembrane system (4%; GO:0012505). The count of other cellular component annotations was below 4%. 

As shown in Figure 4b, the distribution of the cellular component annotations of the top 25 most abundant proteins was slightly different from the annotations of all proteins. A supramolecular complex (11%; GO:0099080) was the third-largest class of annotations after intracellular organelle (14%; GO:0043229) and organelle (14%; GO:0043226). Furthermore, sarcomere GO terms (8%; GO:0030017) were of higher abundance, while cytosol (3%; GO:0005829) and membrane-enclosed lumen (2%; GO:0031974) annotations were less represented than in the case of all proteins for cellular component annotations. Other differences in the distribution of cellular component annotations were smaller and are displayed in Figure 4.

### 3.3. Identification and Characterization of Detected A. simplex Allergens and Potential Allergens 

Among the identified, the following A. simplex allergens were found: Ani s 1, Ani s 2, Ani s 3, Ani s 4, and Ani s 5. We found 13 potential allergens using the AllergenOnline.org server. All detected allergens and potential allergens are listed in Table 2 and Table 3, respectively. 

Table 4 shows all identified peptides of Ani s 1 and Ani s 5 and matched with these peptides the experimentally verified epitopes from IEDB. B-cell and T-cell epitopes were found in all six peptides of Anis s 1, while B-cell epitopes were detected in 8 of 13 peptides of Ani s 5. Experimentally confirmed epitopes in Ani s 2, Ani s 3, and Ani s 4 were not found in IEDB. In silico predicted epitopes in Ani s 2, Ani s 3, and Ani s 4 peptides are shown in Table 5. Among all 74 Ani s 2 peptides, MHC II, T-cell, and B-cell epitopes were predicted in 15, 31, and 10 peptides, respectively. Ten and nine of all 34 Ani s 3 peptides were matched with T-cell and B-cell epitopes, respectively. T-cell epitopes were found in all five peptides of Ani s 4. 

**Figure 5:**
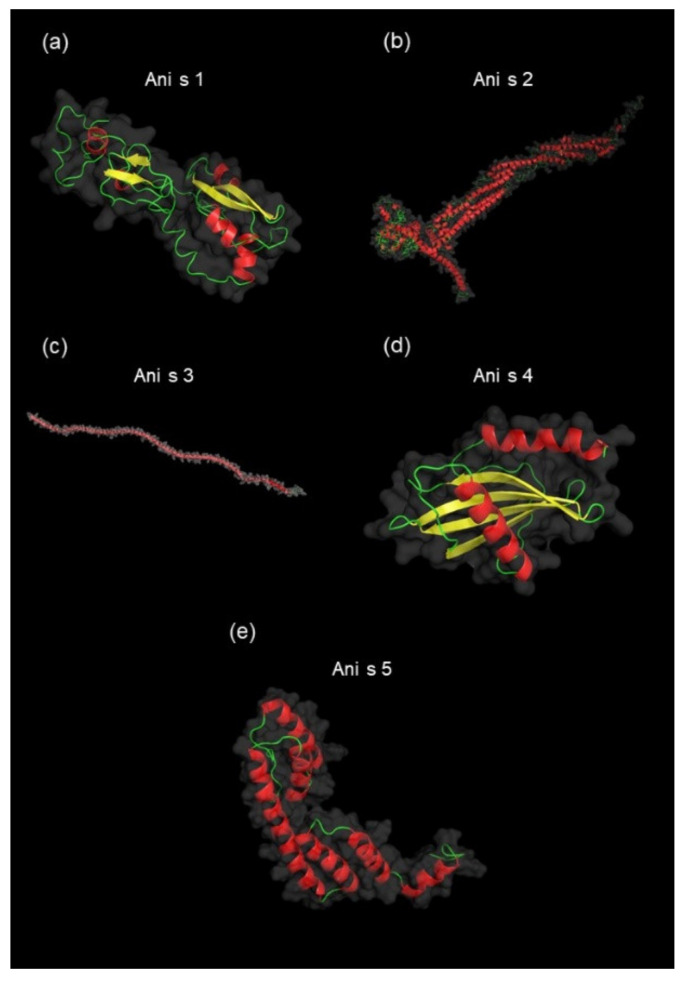
The native tertiary structures of identified allergens. Prediction of unknown 3D structures of allergens: Ani s 1 (77% of residues modeled at > 90% confidence, model dimensions in angstroms (Å): X:64.335 Y:49.060 Z:49.310) (**a**), Ani s 2 (99% of residues modeled at > 90% confidence, model dimensions in Å: X:160.179 Y:213.187 Z:160.224) (**b**), Ani s 3 (99% of residues modeled at > 90% confidence, model dimensions in Å: X:368.887 Y:109.731 Z:146.232) (**c**), and Ani s 4 (86% of residues modeled at > 90% confidence, model dimensions in Å: X:31.951 Y:46.659 Z:32.835) (**d**) were performed using the Phyre2 server. The allergen Ani 5 structure was visualized based on the structure from the Protein Data Bank PDB ID: 2MAR (**e**). PyMOL software was used to visualize all structures. Colors of the allergen models are according to secondary structure: helices highlighted in red, sheets in yellow, and loops in green.

Additionally, the native tertiary structures of the identified allergens were modeled to obtain structural insights into the characteristics of the proteins. Figure 5 shows the native 3D structures of Ani s 1, Ani s 2, Ani s 3, and Ani s 4 modeled with high confidence (≥ 77% of residues modeled at > 90% confidence) using the Phyre2 server and the structure of Ani s 5 derived from RCSB PDB (ID: 2MAR). The displayed structures clearly differentiated various protein and allergen classes.

**Figure 6:**
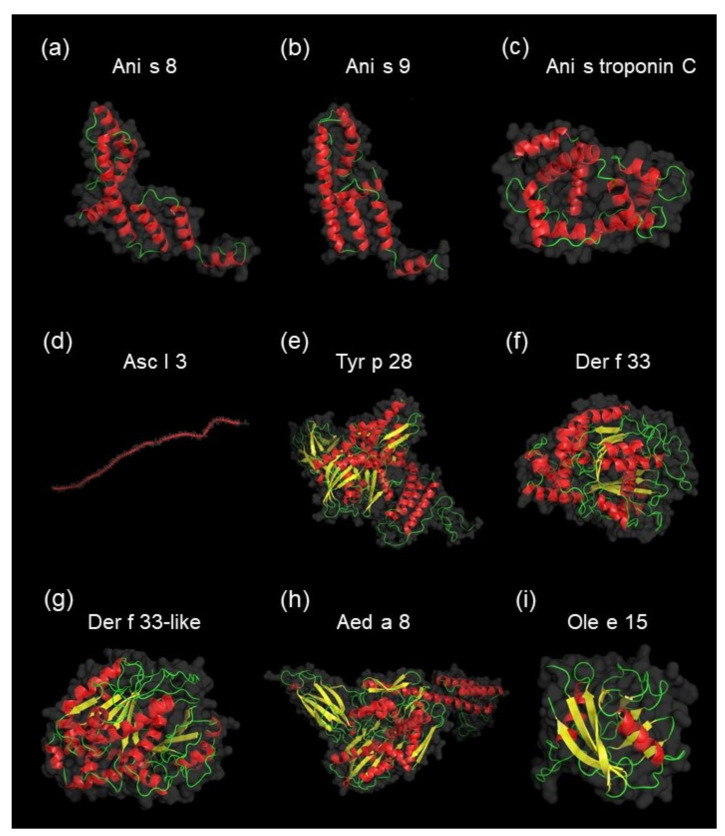
The native tertiary structures of homologous allergens, as identified using AllergenOnline.org for potential allergens of *A. simplex*. Prediction of unknown 3D structures of allergens: Ani s 8 (85% of residues modeled at > 90% confidence, model dimensions in angstroms (Å): X: 62.875 Y: 60.531 Z: 35.486) (**a**), Ani s 9 (87% of residues modeled at > 90% confidence, model dimensions in Å: X:54.278 Y: 58.349 Z:28.680) (**b**), Ani s troponin C (99% of residues modeled at > 90% confidence, model dimensions in Å: X:50.390 Y: 40.831 Z:35.415) (**c**), Asc l 3 (99% of residues modeled at > 90% confidence, model dimensions in Å: X:361.607 Y:132.976 Z:152.396) (**d**), Tyr p 28 (97% of residues modeled at > 90% confidence, model dimensions in Å: X:81.072 Y:91.096 Z:63.286) (**e**), Der f 33 (89% of residues modeled at > 90% confidence, model dimensions in Å: X:63.736 Y:59.230 Z:58.888) (**f**), Der f 33-like (90% of residues modeled at > 90% confidence, model dimensions in Å: X:57.763 Y:55.796 Z:56.582) (**g**), Aed a 8 (90% of residues modeled at > 90% confidence, model dimensions in Å: X:57.763 Y:55.796 Z:56.582) (**h**), Ole e 15 (100% of residues modeled at > 90% confidence, model dimensions in Å: X:38.017 Y:36.365 Z:47.475) (**i**) were performed using the Phyre2 server. Allergen 3D structures of Ani s 2 and Ani s 5, which are also homologs of potential allergens, are shown in Figure 5. PyMOL software was used to visualize all structures. Colors of the allergen models are according to secondary structure: helices highlighted in red, sheets in yellow, and loops in green.

The largest group of potential allergens (five proteins) were homologous to A. simplex allergens (Ani s 2, Ani s 5, Ani s 8, Ani s 9, and Ani s troponin C). Four potential allergens were homologous to the following Acari (Tyrophagus putrescentiae, Dermatophagoides farina, Dermatophagoides pteronyssinus) allergens: Tyr p 28, Der f 33, and Der f 33-like. Another two proteins were homologous to Ascaris lumbricoides allergen Asc l 3. The last two potential allergens of A. simplex were homologous to Olea europaea allergen Ole e 15 and Aedes aegypti allergen Aed a 8. Three-dimensional native structures of homologous allergens for potential allergens of A. simplex are shown in Figure 6 (≥ 85% of residues modeled at > 90% confidence), except for the Ani s 2 and Ani s 5 models, which are presented in Figure 5. Additionally, the alignment of 3D models of potential allergens and their homologous allergens was performed to visualize the similarities in protein structures (see Supplemental File S2). The displayed 3D models of allergens and potential allergens showed similarities among the proteins belonging to the same allergen classes.

*A. simplex* allergens and homologs of potential allergens (see Table 2 and Table 5) were classified to allergen families based on the AllFam database. The five following AllFam families were detected among identified allergens: animal Kunitz serine protease inhibitor (Ani s 1), myosin heavy chain (Ani s 2), tropomyosin (Ani s 3), cystatin (Ani s 4), and SXP/RAL-2 family (Ani s 5). In the case of homologs of potential allergens, six families of AllFam were found. The most highly represented AllFam families of homologous allergens were heat shock protein 70 (Hsp70) and SXP/RAL-2 family (three matches for each), followed by tropomyosin (two matches). The lowest abundant allergen families (one match for each) were the following: EF-hand family, myosin heavy chain, and tubulin/FtsZ family. Two homologous allergens (Ole e 15 and Der f 33-like) were not reported in the AllFam database.

The detected allergens were in the Mw/pI range of 10291-100461 Da/4.68-7.48, while potential allergens were in the range of 9756-101686 Da/4.15-9.04. The lowest Mw of the detected allergens and potential allergens was found for the Ani s 4 and uncharacterized protein (UniProt ID: A0A0M3J8H0), respectively. Ani s 2 (paramyosin) was the allergen with the highest calculated Mw, while the highest Mw value of potential allergens was found for the paramysoin (UniProt ID: A0A158PP35). Ani s 3 (tropomyosin) and troponin-like protein (UniProt ID: A0A0M3JU57) had the lowest calculated pI values of allergens and potential allergens, respectively. Ani s 1 was the allergen with the highest pI, whereas the highest pI of potential allergens was calculated for the peptidyl-prolyl cis-trans isomerase (UniProt ID: A0A0M3J6G4).

The identified allergens were in the range of emPAI values from 2.53 (Ani s 1) to 62.69 (Ani s 3); while the potential allergens were in the range from 0.42 (tubulin alpha chain; UniProt ID: A0A0M3K821) to 109.71 (tropomyosin; UniProt ID: A0A0M3KCE6). In the top 25 most abundant proteins (see Table 1), the following allergens were found: Ani s 2, Ani s 3, and Ani s 5, and the following possible allergens: tropomyosin (UniProt ID: A0A0M3KCE6), SXP/RAL-2 family protein 2 isoform 1 (UniProt ID: A0A0M3KA05), and paramyosin (UniProt ID: A0A158PP35). The distribution of the detected allergens and potential allergens based on the Mw, pI, and emPAI values is displayed in Figure 2.

All five detected allergens were classified using InterPro analysis, whereas the three following potential allergens: uncharacterized protein (UniProt ID: A0A0M3J8H0), tubulin alpha chain (UniProt ID: A0A0M3K821), and tubulin alpha chain (UniProt ID: A0A0M3KAH2) had no domains or families matches. Among the identified allergens, two InterPro families (pancreatic trypsin inhibitor Kunitz domain superfamily and tropomyosin) and four InterPro domains (pancreatic trypsin inhibitor Kunitz domain, myosin tail, cystatin domain, and domain of unknown function DUF148) were detected. Six InterPro families of potential allergens were found, from which the most abundant (three matches for each) were the heat shock protein 70 family and heat shock protein 70 kD peptide-binding domain superfamily. Less represented (two matches for each) were the following InterPro families: tropomyosin and heat shock protein 70 kD C-terminal domain superfamily, while the least abundant (one match for each) were EF-hand domain pair and cyclophilin-like domain superfamily. Five domains of all possible allergens were identified. The most abundant was the domain of unknown function DUF148 (two matches), and other domains (one match for each) were the following: myosin tail, cyclophilin-type peptidyl-prolyl cis-trans isomerase domain, EF-hand domain, and endoplasmic reticulum chaperone binding immunoglobulin protein (BIP) nucleotide-binding domain. 

**Figure 7:**
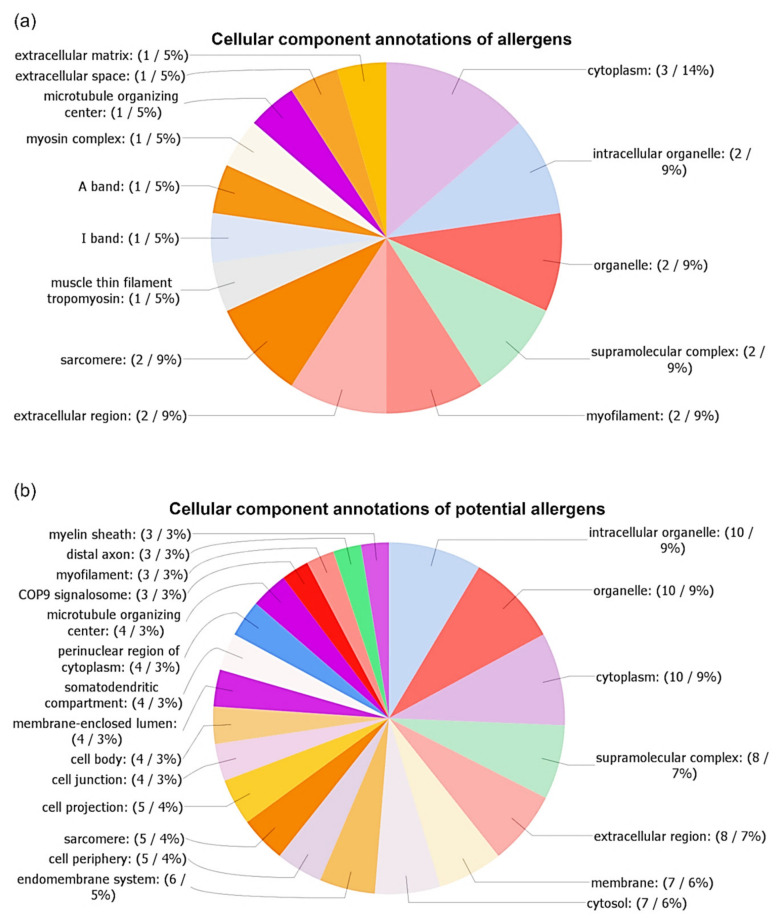
Gene ontology analysis of cellular components of allergens (**a**) and potential allergens (**b**). The number of annotations was limited for better readability in the case of potential allergens (minimum of three sequences). Annotations were performed using OmicsBox software.

The distribution of cellular component annotations of allergens and potential allergens are displayed in Figure 7a,b, respectively. A detailed list of all GO terms with assigned sequences is shown in Supplemental File S1. The most abundant class of cellular component annotations of allergens was cytoplasm (14%; GO:0005737), with three matched sequences: Ani s 2, Ani s 3, and Ani s 5. Lower represented (9%) were the following annotations: intracellular organelle (matches: Ani 2, Ani s 3; GO:0043229), organelle (matches: Ani s 2, Ani s 3; GO:0043226), supramolecular complex (matches: Ani s 2, Ani s 3; GO:0099080), myofilament (matches: Ani s 2, Ani s 3; GO:0036379), extracellular region (matches: Ani s 1, Ani s 5; GO:0005576), and sarcomere (matches: Ani s 2, Ani s 3; GO:0030017). The least abundant (5%) cellular component annotations were muscle thin filament tropomyosin (match: Ani s 3; GO:0005862), I band (match: Ani s 3; GO:0031674), A band (match: Ani s 2; GO:0031672), myosin complex (match: Ani s 2; GO:0016459), microtubule organizing center (match: Ani s 2; GO:0005815), extracellular space (match: Ani s 5; GO:0005615), and extracellular matrix (match: Ani s 1; GO:0031012).

Twenty-one cellular components GO terms were found based on the analysis of potential allergens. The most abundant of them were the following GO terms: intracellular organelle (9%; GO:0043229), organelle (9%; GO:0043226), cytoplasm (9%; GO:0005737), followed by supramolecular complex (7%; GO:0099080), extracellular region (7%; GO:0005576), membrane (6%; GO:0016020), and cytosol (6%; GO:0005829). Other cellular components GO terms were below 6%.

## 4. Discussion

The sterilization process is widely used for preserving many types of food, including products containing the meat of sea fish. Studies evaluating the effects of such a process on the *A. simplex* proteins, which could contaminate fish products, are only fragmentary. Therefore, in the present work, we attempted to reduce the knowledge gap in this topic, especially focusing on the proteome of *Anisakis*. A current survey was the first proteome and allergome profiling of *A. simplex* in autoclaving conditions.

### 4.1. Electrophoretic and Immunological Investigations of the Influence of High Temperature on A. simplex Antigen

We performed a comparative analysis of SDS-PAGE and IgG-WB profiles of the following *A. simplex* antigens: (i) native antigen, (ii) antigen heated at 100 °C, and (iii) autoclaved antigen. We showed that antigen heating and autoclaving caused a reduction in the number and intensity of SDS-PAGE and IgG-WB bands. Furthermore, both heating processes caused an appearance of background in IgG-WB profiles. Based on this experiment, we knew that heating caused changes in the antigens, but did not interfere with the ability to bind antibodies. Similar observations regarding the reduction of the number and intensity of bands in electrophoretic and WB profiles of autoclaved *Anisakis* antigen were reported by Carballeda-Sangiao et al. [51] and Klapper et al. [52]. 

We thought that the background in the IgG-WB profiles of the heated antigens was the result of degradation and/or alteration of some of the *Anisakis* proteins. However, heated antigens were degraded only partially. Similarly to our study, several other investigations concerning the influence of autoclaving on antigens/allergens of tree nuts [53], shrimps [54], lentil, and chickpeas [55] have shown smear appearing on the SDS-PAGE and WB profiles while maintaining their antigenicity.

Based on the calculation of the IntDen values of the SDS-PAGE and IgG-WB profiles, we measured the protein amounts in the antigens and the signal intensity of the immunoreactivity of antigens, respectively. The IntDen values of SDS-PAGE, as well as the IgG-WB profiles, were similar for the native and both heated antigens; therefore, we supposed that the temperature conditions we used did not cause a drastic reduction of antigenicity. 

The present study confirmed the immunoreactivity of the heated/autoclaved *Anisakis* antigens with anti-*A. simplex* IgG antibodies that were linked to delayed IgG-based allergy. This type of hypersensitivity is associated with chronic urticaria that is a frequent symptom of anisakiasis [56,57]. Detection of IgG antibodies specific to *Anisakis* is also useful in the serodiagnosis of anisakiasis [42]. The IgE immunoreactivity of heated *Anisakis* antigens was not investigated in this study, and further studies on this issue are needed. However, the IgE immunoreactivity and thermostability of autoclaved Ani s 1 and Ani s 4 have been shown by Carballeda-Sangiao et al. [51]. Results of other studies that investigated the influence of high temperature on the antigen of *A. simplex* [51,52,58,59,60,61] were consistent with our findings regarding the high thermal resistance of *Anisakis* antigenic profile.

### 4.2. Identification and Label-Free Quantification of Proteins, Allergens, and Potential Allergens of A. simplex 

Mass spectrometry allowed us to detect 470 proteins of *A. simplex*. Another published dataset of proteins derived from heat-treated *Anisakis* larvae consists of 146 proteins detected in nematode extract heated 5 min at 110 °C [62]. There are no other thermal proteome profiles of *A. simplex* reported in the scientific literature. Comparing the total number of detected proteins (*n* = 470) from the present study with the total number of proteins of native *Anisakis* antigen from our previous investigation [23], we could see a 27% decrease in protein number after autoclaving. In the proteomic profiling of native antigens of *A. simplex* [23], we used a slightly different bioinformatic approach; nevertheless, this comparison clearly indicated that autoclaving did not drastically reduce the number of proteins.

Among all the detected proteins, we identified peptides derived from the five following allergens of *A. simplex*: Ani s 1, Ani s 2, Ani s 3, Ani s 4, and Ani s 5. However, it was impossible to confirm the thermostability of the detected allergens since the identification was conducted by the gel-free LC-MS/MS approach, and, using this technology, only peptides could be detected. However, epitopes, as well as possible epitopes, were found in identified allergenic peptides. As was mentioned, among the *A. simplex* allergens from the WHO/IUIS list, until now, only the autoclaving resistance of Ani s 1 and Ani s 4 is known [51], and further thermostability studies of other *Anisakis* allergens are necessary. The total number of allergens detected in the autoclaved antigens (*n* = 5) was 64% lower compared to the number of allergens found in the native *Anisakis* antigen (*n* = 14) [23]. However, it should be emphasized that, as identified in our present study, Ani s 1 and Ani s 2 are particularly harmful as they are major allergens, which cause hypersensitivity reactions in more than 50% of the allergic population [63]. Ani s 2 and Ani s 3 are also panallergens that are ubiquitously distributed with highly conserved sequences and structures, and, therefore, they are responsible for cross-reactions, even between phylogenetically distant and unrelated organisms [64,65,66]. 

In recent studies, novel allergens and novel potential allergens of *Anisakis* have been described. As the list of *A. simplex* allergens seems to be still incomplete, we performed bioinformatic screening of the detected proteins for possible allergenicity. We identified 13 potential allergens of *Anisakis* (see Table 5) using the AllergenOnline.org server, which is commonly used for such computational evaluations [67]. To increase the confidence of the allergenicity predictions, we applied a high level of identity between potential allergens and homologous allergens (70%) as the cut-off, as the level of identity already just above 50% indicates the possibility of cross-reactions [68]. Most of the homologous identified potential allergens were allergens of *Anisakis* (*n* = 5) and *Ascaris* (*n* = 2), which are relative phylogenetic closely related as both nematodes belong to the same order of Ascaridida. Among the potential allergens, we also frequently detected homologs of Acari allergens (*n* = 4). The occurrence of cross-reactions of *A. simplex* antigen with Acari [69] and *Ascaris* [70,71] antigens was experimentally proven.

Three following potential allergens identified in this work: paramyosin (UniProt ID: A0A158PP35), peptidyl-prolyl cis-trans isomerase (UniProt ID: A0A0M3J6G4), and 78 kDa glucose-regulated protein (UniProt ID: A0A0M3K5H6) were also detected by us in the native antigens of *A. simplex* [23]. We identified potential *Anisakis* allergen SXP/RAL-2 family protein 2 isoform 1 (UniProt ID: A0A0M3KA05) in the native antigen of *Contracaecum osculatum* [23]. Except for these four potential allergens identified in our previous study, the other nine sequences were identified as potential allergens of *Anisakis* for the first time. 

To know the content of allergens and potential allergens in heat-treated *A. simplex* larvae, we measured the relative abundance of proteins using mass spectrometry label-free quantification. We analyzed the protein abundance calculated by Mascot software, and this data revealed that among the 25 most abundant proteins were present the following allergens: Ani s 2, Ani s 3, and Ani s 5, and the following potential allergens: tropomyosin (UniProt ID: A0A0M3KCE6), SXP / RAL-2 family protein 2 isoform 1 (UniProt ID: A0A0M3KA05), and paramyosin (UniProt ID: A0A158PP35). Comparing these results with the relative quantification of allergens found among the 25 most abundant proteins of the native antigen of *Anisakis* [23], we could conclude that autoclaving caused a slight reduction in the number of identified allergens. The following five allergens were found in the native antigens: Ani s 8, Ani s 2, Ani s 13, and Ani s 3 (two isoforms). 

### 4.3. Computational Investigations of Detected Proteins, Allergens, and Potential Allergens of A. simplex

Especially important bioinformatics analysis was the detection of epitopes in allergenic peptides originated from the autoclaved antigen of *A. simplex*. This investigation confirmed the results of WB and showed that the epitopes of autoclaved peptides were not destroyed. Among all *Anisakis* allergens, T-cell/B-cell epitopes in Ani s 1 [48,49] and Ani s 5 [50] were experimentally verified, and we used these datasets via IEDB. Epitopes were identified in the majority (74%) of Ani s 1 and Ani s 5 peptides (see Table 3). MHC II, T-cell, and B-cell epitopes in Ani s 2, Ani s 3, and Ani s 4 were predicted for the first time. Probable epitopes were found in 61% of autoclaved allergenic peptides (Table 4) using algorithms of DNASTAR Protean 3D software. This software has been successfully used to predict epitopes in many other studies [72,73,74].

Previously, among all *Anisakis* allergens, the only 3D structure of Ani s 5 has been determined experimentally by nuclear magnetic resonance [50]. Therefore, to acquire more insights into the nature of novel potential allergens, as well as all identified allergens, we predicted 3D models, representing the native conformation of these proteins. The models were predicted with high confidence using an algorithm combining ab initio and homology-based prediction implemented in the Phyre2 server [47]. Pairs of protein models representing the potential allergen and its homologs were subjected to structural alignment within the PyMOL software. Structural alignment analysis confirmed a potential allergen’s structural similarities with its homologs.

Computational analyses showed that identified proteins, allergens, and potential allergens were very diverse in properties, such as molecular weights and isoelectric points. High diversity also occurred at the level of protein classification into the InterPro family, the identification of InterPro domains, and allergen assignment according to the AllFam database. For example, the most highly represented family and domain among all detected proteins, i.e., immunoglobulin-like fold (IPR013783) and immunoglobulin-like domain (IPR007110) were assigned only to 4% and 3% of sequences, respectively. This superfamily represents domains with an immunoglobulin-like (Ig-like) fold, and Ig-like domains are one of the most common protein modules found in different organism proteins [75]. Proteins with this fold vary in their cellular localization, amino acid sequence, and biological role [76]. EF-hand domains were relatively abundant among identified proteins (nine sequences). Troponin-like protein (UniProt ID: A0A0M3JU57), a potential allergen detected in this study, and its homolog (Ani s troponin C) also contain EF-hand domains. EF-hand allergen family is the second-largest group of allergens [77] that are detected in different organisms like parvalbumins in a specific species of fish and fungus (*Trichophyton violaceum*) [78] or polcalcins in the pollen of trees, grasses, and weeds [79]. It has been found that antigenic sites of parvalbumins in *Trichophyton violaceum* are located on both sides of the Ca^2+^-binding site of the first EF-hand domain and parvalbumin proteins possessing conserved amino acid motifs (cysteine, lysine, and arginine) [78]. Noteworthy proteins among the highly abundant proteins families detected by us were heat shock proteins, like HSP20-like chaperones (eight sequences), which were highly represented among all thermostable proteins, while hsp70s were relatively higher abundant (three sequences) in the group of potential allergens. In our previous study, we found that heat shock proteins were also one of the most abundant proteins in the native antigen of *Anisakis* [23]. This is not surprising because HSPs are extremely heterogeneous in nature and function mainly as molecular chaperones that help other proteins maintain their native structure and, especially under stresses, are highly expressed [80,81,82]. HSPs are major immune dominant antigens in many parasite infections, and they play a key role in host–parasite interactions [83,84]. Allergens belonging to the Hsp70 family are found in a heterogeneous range of sources. Among others, HSP70s are inhalant allergens of house dust mites, storage mites, biting midges, black flies, and cockroaches [20,85,86]. 

AllFam classification is an effective way to characterize allergens and potential allergens. This was also the case in our study in which, despite a large variety of detected allergen, the following classes were more common: hsp70 (AF002), which is described above, SXP/RAL-2 family (AF137), tropomyosin (AF054), and myosin heavy chain (AF100). 

The allergens of the SXP/RAL-2 family include only three allergens: Ani s 5, Ani s 7, and Ani s 8. In addition to the Ani s 5 allergen, in the present study, we identified two potential allergens related to this family. Members of the SXP/RAL-2 family are characterized by the presence of the domain of unknown function (DUF)148 protein [87]. This family of secreted proteins seems to be specific for nematodes, and several members have been reported in animal parasitic nematodes and in *Caenorhabditis elegans* [87,88,89]. The role of these proteins is unrecognized; however, it is known that the structure of Ani s 5 resembles that of calmodulin but binds Mg^2+^ instead of Ca^2+^ [50].

Among allergens from the tropomyosin class, we found Ani s 3 and two potential allergens that were homologs of Asc l 3. Tropomyosin has been identified as a minor inhalation allergen in arthropods (mites, cockroaches) and as a major food allergen in crustaceans and mollusks [90,91], while vertebrate tropomyosin seems to be non-allergenic [92]. Due to repetitive coiled-coil structures, which were visualized on our 3D models, tropomyosin retains IgE antibodies binding ability even after heat treatment or partial digestion [92,93]. Tropomyosin sequences are highly conserved, which causes frequent cases of hypersensitivity cross-reactions with phylogenetically distant allergens [94].

During the analysis of allergen class of myosin heavy chain (AF100), we assigned Ani s 2 and one potential homolog allergen of Ani s 2 to this group. Both of these proteins were paramyosins. Paramyosin is a filamentous protein that is found in many invertebrates, including parasites. This protein may regulate the host’s immune responses by inhibiting the classical pathway of complement cascade through inhibition of the complement C1 function [95]. Paramyosin is engaged in the immunological protection mechanism of parasites by acting as Fc receptors and has been shown to induce hypersensitivity reactions in humans [96,97,98]. 

We performed cellular component GO annotation to predict the localization of detected proteins in relation to cellular compartments and structures. Obtaining this data allowed for a deeper characterization of proteins as it provided context enabling, understanding of their function. The results of the cellular components annotations (see Figure 4 and Figure 7) showed a large variation in the distribution of GO terms. Among all annotations, the most abundant (about one-third of annotations) were the following GO terms: organelle (GO:0043226), intracellular organelle (GO:0043229), and cytoplasm (GO:0005737). The GO term organelle means the organized structure of distinctive morphology and function, includes the nucleus, mitochondria, vesicles, ribosomes, and the cytoskeleton, but excludes the plasma membrane. The GO term intracellular organelle is an organized structure of distinctive morphology and function, occurring within the cell. The GO term cytoplasm is all of the contents of a cell, excluding the plasma membrane and nucleus, but including other subcellular structures.

In our comparison of the cellular component annotations among all identified proteins, the most abundant 25 proteins, allergens, and potential allergens showed some interesting differences. The GO term supramolecular complex (GO:0099080) was the most represented in the 25 most abundant proteins (11% of sequences), followed by allergens (9% of sequences), potential allergens (7% of sequences), and the lowest represented in case of all proteins (5%). The GO term supramolecular complex is a cellular component that consists of an indeterminate number of proteins or macromolecular complexes, organized into regular, higher-order structures, such as a polymer, sheets, networks, or fibers. Among the proteins belonging to this class of GO term, we also assigned the following allergens and potential allergens, which were filament proteins: tropomyosin (including Ani 3), paramyosin (including Ani s 2), tubulin alpha chain, and troponin-like protein sequences. Slightly higher abundance of sequences of allergens, as well as potential allergens, were assigned to the extracellular region (GO:0005576). The GO term extracellular region is the space external to the outermost structure of a cell; for cells without external protective or external encapsulating structures, this refers to space outside of the plasma membrane. The following proteins were assigned to this GO term: like tubulin alpha chain, SXP/RAL-2 family protein, 78 kDa glucose-regulated protein, DUF148 domain-containing protein, and heat shock 70 kDa protein cognate 1. These results corresponded with the fact that Ani s 1 and Ani s 5 are excretory-secretory allergens.

## 5. Conclusions

This study provided novel data on the *A. simplex* proteome. Based on mass spectrometry analysis, it could be concluded that 470 proteins were detected in heat-treated *A. simplex* larvae. Among identified proteins, peptides of the following allergens were found: Ani s 1, Ani s 2, Ani s 3, Ani s 4, and Ani s 5. In silico predicted and known epitopes in peptides originated from these allergens were detected using bioinformatics tools. Furthermore, thirteen potential allergens were detected, nine of which were identified for the first time. The identified proteins, allergens, and potential allergens were very diverse in terms of properties, such as their molecular weight, isoelectric point, tertiary structure, domain and family classifications, and cellular component annotations. The reactivity of the autoclaved *A. simplex* antigen with anti-*A. simplex* IgG antibodies that were relevant to delayed IgG-based allergy was confirmed by WB. The IgE-binding capacity and thermostability of identified allergens and potential allergens were not tested in this study, and, therefore, further studies are needed to investigate these aspects.

Due to the presence of epitopes in allergenic peptides derived from the autoclaved antigen, thermally processed fish products that might contain *A. simplex* proteins could be a potential threat to sensitized consumers. These findings have implications for the fish processing industry and food safety authorities. It is necessary to search for more effective methods to reduce the allergenicity of food products contaminated by *Anisakis*. A practical solution to this issue can provide removal of *Anisakis* allergens during the washing of fish muscle, as described by Olivares et al. [99]. Furthermore, an extensive examination of fish products for *A. simplex* allergens can improve the protection of *Anisakis*-allergic consumers. Hence, the implementation of diagnostic tools for the detection of *A. simplex* allergens is essential for food safety laboratories. Publicly deposited mass spectrometry data could be useful for future studies, such as the development of new diagnostic assays. 

## Figures and Tables

**Table 1:** The top 25 most abundant proteins of *Anisakis simplex* identified by liquid chromatography-tandem mass spectrometry (LC-MS/MS).

No	UniProt ID	Protein Name ^1^	InterPro Family ^2^ (ID)	InterPro Domain ^2^ (ID)	Mw ^3^ (Da)	pI ^3^	Mascot Scor ^4^	Coverage ^4^ (%)	emPAI Value ^4^
1	A0A0M3K6N3	Myosin, essential light chain (inferred by orthology to a Caenorhabditis elegans protein)	EF-hand domain pair (IPR011992); Myosin light chain alkali (IPR029655)	EF-hand domain (IPR002048)	16,418	4.89	4418	90	201.76
2	A0A0M3KCE6	Tropomyosin (inferred by orthology to a C. elegans protein)	Tropomyosin (IPR000533)	-^5^	13,701	4.54	3023	63	109.71
3	A0A0M3J349	DUF4440 domain-containing protein	Nuclear transport factor 2 (NTF2)-like domain superfamily (IPR032710)	Domain of unknown function DUF4440 (IPR027843)	13,671	6.29	785	72	81.05
4	A0A0M3J4D2	Uncharacterized protein	-	-	23,930	8.72	1897	71	67.66
5	A0A0M3K7H9	Uncharacterized protein	-	-	11,337	9.89	922	70	63.02
6	Q9NAS5	Tropomyosin (allergen Ani s 3)	Tropomyosin (IPR000533)	-	33,203	4.68	3241	72	62.69
7	A0A0M3KA05	SXP/RAL-2 family protein 2 isoform 1	-	Domain of unknown function DUF4440 (IPR027843)	14,517	4.52	5725	71	45.10
8	A0A0M3K8L6	Uncharacterized protein	DVA-1 superfamily (IPR038289)	Polyprotein allergen, nematode (IPR032487)	42,337	7.07	3504	62	42.49
9	A0A0M3KF53	Uncharacterized protein	-	-	26,477	4.98	2630	70	38.24
10	A0A0M3KIS5	IF rod domain-containing protein	Intermediate filament, rod domain, coil 1B (IPR042180)	Intermediate filament, rod domain (IPR039008)	27,871	5.17	1999	73	33.77
11	A0A0M3JCV3	KASH_CCD domain-containing protein	-	KASH5-like coiled-coil domain (IPR028168)	17,230	5.4	1885	68	32.84
12	A0A0M3J6C9	Uncharacterized protein	-	-	26,754	7.78	1755	54	30.87
13	A0A0M3JGD2	Uncharacterized protein	-	-	10,889	4.86	771	52	23.42
14	A0A158PP35	Paramyosin (inferred by orthology to a C. elegans protein)	-	Myosin tail (IPR002928)	101,686	5.34	11203	67	22.63
15	A0A0M3JX08	Protein lethal(2)essential for life (inferred by orthology to a Drosophila melanogaster protein)	HSP20-like chaperone (IPR008978); Alpha-crystallin/Heat shock protein (IPR001436)	Alpha-crystallin/Hsp20 domain (IPR002068)	21,063	5.98	1284	80	21.20
16	A0A0M3JAH1	Intermediate filament protein ifb-1 (inferred by orthology to a C. elegans protein)	Lamin tail domain superfamily (IPR036415)	Intermediate filament, rod domain (IPR039008); Lamin tail domain (IPR001322)	24,324	5.33	991	65	20.01
17	A0A0M3JTQ4	Myosin regulatory light chain 2 (inferred by orthology to a D. melanogaster protein)	EF-hand domain pair (IPR011992)	EF-hand domain (IPR002048)	18,715	5.05	2597	81	19.51
18	A0A0M3KKR0	Uncharacterized protein	-	-	5009	4.79	1077	56	18.10
19	A0A0M3J766	Uncharacterized protein	-	-	13,471	5.82	522	48	16.84
20	Q9NJA9	Paramyosin (allergen Ani s 2)	-	Myosin tail (IPR002928)	100,461	5.21	9369	69	16.04
21	A1IKL2	SXP/RAL-2 family protein Ani s 5 (allergen Ani s 5)	-	Domain of unknown function DUF4440 (IPR027843)	14,738	4.85	1765	47	14.65
22	A0A0M3JID9	IF rod domain-containing protein	Intermediate filament, rod domain, coil 1B (IPR042180)	Intermediate filament, rod domain (IPR039008)	10,555	6.29	649	43	14.45
23	A0A0M3JGV0	Uncharacterized protein	-	-	13,766	4.73	844	42	14.31
24	A0A0M3IYQ5	Glutamate dehydrogenase, mitochondrial (inferred by orthology to a D. melanogaster protein)	NAD(P)-binding domain superfamily (IPR036291)	-	11,634	8.89	650	61	14.09
25	A0A0M3J159	Small heat shock protein (SHSP) domain-containing protein	HSP20-like chaperone (IPR008978); Alpha-crystallin/Heat shock protein (IPR001436)	Alpha-crystallin/Hsp20 domain (IPR002068)	13,230	6.29	1036	54	13.78

^1^ Protein name according to the Universal Protein Resource (UniProt); ^2^ InterPro families and InterPro domains were predicted using OmicsBox software; ^3^ the theoretical molecular weight (Mw) in daltons (Da) and theoretical isoelectric point (pI) were estimated using the ExPASy Compute pI/Mw tool; ^4^ Mascote score, coverage (in %), and the exponentially modified protein abundance index (emPAI) were calculated as the average of three biological replicates using values derived from Mascot; ^5^ not detected.

**Table 2:** Allergens of *A. simplex* identified using LC-MS/MS.

No	UniProt ID	Allergen Name ^1^	Protein Name	InterPro Family (ID)	InterPro Domain (ID)	AllFam ^2^ (ID)	Mw (Da)	pI	Mascot Score	Coverage (%)	emPAI Value
1	Q7Z1K3	Ani s 1	Major allergen Ani s 1 (21 kDa allergen)	Pancreatic trypsin inhibitor Kunitz domain superfamily (IPR036880)	Pancreatic trypsin inhibitor Kunitz domain (IPR002223)	Animal Kunitz serine protease inhibitor (AF003)	18,869	7.48	481	21	2.53
2	Q9NJA9	Ani s 2	Paramyosin	-	Myosin tail (IPR002928)	Myosin heavy chain (AF100)	100,461	5.21	9369	69	16.04
3	Q9NAS5	Ani s 3	Tropomyosin	Tropomyosin (IPR000533)	-	Tropomyosin (AF054)	33,203	4.68	3241	72	62.69
4	Q14QT4	Ani s 4	Ani s 4 allergen	-	Cystatin domain (IPR000010)	Cystatin (AF005)	10,291	5.54	196	27	3.67
5	A1IKL2	Ani s 5	SXP/RAL-2 family protein Ani s 5	-	Domain of unknown function DUF148 (IPR003677)	SXP/RAL-2 family (AF137)	14,738	4.85	1765	47	14.65

^1^ Allergen name approved by the World Health Organization and International Union of Immunological Societies (WHO/IUIS) Allergen Nomenclature Sub-committee; ^2^ classification of allergen family was conducted according to ‘AllFam - The Database of Allergen Families’ (ver. 2017.03.07).

**Table 3:** Potential allergens of *A. simplex* identified by LC-MS/MS and AllergenOnline.org analysis.

	Possible Allergens ^1^							Homologous Allergens ^1^				AllergenOnline.org Result ^1^	
No	UniProt ID	Protein Name	InterPro Family (ID)	InterPro Domain (ID)	Mw (Da)	pI	emPAI Value	Genbank ID	ALLERGEN name	Protein Name (Taxonomy)	AllFam (ID)	Identity ^1^	Similarity ^1^
1	A0A0M3KCE6	Tropomyosin (inferred by orthology to a C. elegans protein)	Tropomyosin (IPR000533)	-	13,701	4.54	109.71	ACN32322.1	Asc l 3	Tropomyosin (Ascaris lumbricoides)	Tropomyosin (AF054)	0.946	0.973
2	A0A0M3KA05	SXP/RAL-2 family protein 2 isoform 1	-	Domain of unknown function DUF148 (IPR003677)	14,517	4.52	45.10	BAF75681.1	Ani s 8	SXP/RAL-2 family protein 2 isoform 1 (Anisakis simplex)	SXP/RAL-2 family (AF137)	0.987	1
3	A0A158PP35	Paramyosin (inferred by orthology to a C. elegans protein)	-	Myosin tail (IPR002928)	101,686	5.34	22.63	Q9NJA9.1	Ani s 2	Paramyosin (Anisakis simplex)	Myosin heavy chain (AF100)	0.854	0.958
4	A0A0M3J4T5	DUF148 domain-containing protein	-	Domain of unknown function DUF148 (IPR003677)	16,646	5.56	7.10	BAF43534.1	Ani s 5	SXP/RAL-2 family protein (Anisakis simplex)	SXP/RAL-2 family (AF137)	0.971	0.993
5	A0A0M3J693	Tropomyosin (inferred by orthology to a C. elegans protein)	Tropomyosin (IPR000533)	-	11,304	4.64	7.79	ACN32322.1	Asc l 3	Tropomyosin (Ascaris lumbricoides)	Tropomyosin (AF054)	0.824	0.953
6	A0A0M3K9V2	Heat shock 70 kDa protein cognate 1 (inferred by orthology to a D. melanogaster protein)	Heat shock protein 70 kD, peptide-binding domain superfamily (IPR029047); Heat shock protein 70 family (IPR013126); Heat shock protein 70 kD, C-terminal domain superfamily (IPR029048)	-	67,585	5.45	4.84	AOD75395.1	Tyr p 28	Heat shock-like protein (Tyrophagus putrescentiae)	Heat shock protein (Hsp70) (AF002)	0.737	0.86
7	A0A0M3KIW1	Uncharacterized protein	Heat shock protein 70 kD, peptide-binding domain superfamily (IPR029047); Heat shock protein 70 family (IPR013126)	-	24,599	8.49	2.31	AOD75395.1	Tyr p 28	Heat shock-like protein (Tyrophagus putrescentiae)	Heat shock protein (Hsp70) (AF002)	0.851	0.95
8	A0A0M3J6G4	Peptidyl-prolyl cis-trans isomerase	Cyclophilin-like domain superfamily (IPR029000)	Cyclophilin-type peptidyl-prolyl cis-trans isomerase domain (IPR002130)	12,614	9.04	1.99	AVV30163.1	Ole e 15	Cyclophilin 0101 (Olea europaea)	-	0.789	0.916
9	A0A0M3JU57	Troponin-like protein	EF-hand domain pair (IPR011992)	EF-hand domain (IPR002048)	19,444	4.15	1.39	CAB58171.1	Ani s troponin C	Troponin-like protein (Anisakis simplex)	EF hand family (AF007)	0.994	1
10	A0A0M3K5H6	78 kDa glucose-regulated protein	Heat shock protein 70 kD, peptide-binding domain superfamily (IPR029047); Heat shock protein 70 family (IPR013126); Heat shock protein 70 kD, C-terminal domain superfamily (IPR029048)	Endoplasmic reticulum chaperone BIP, nucleotide-binding domain (IPR042050)	75,409	5.07	0.77	ABF18258.1	Aed a 8	Heat shock cognate 70 (Aedes aegypti)	Heat shock protein (Hsp70) (AF002)	0.777	0.93
11	A0A0M3J8H0	Uncharacterized protein	-	-	9756	5.52	0.51	ABV55106.1	Ani s 9	Ani s 9 allergen precursor (Anisakis simplex)	SXP/RAL-2 family (AF137)	1	1
12	A0A0M3K821	Tubulin alpha chain	-	-	47,784	4.83	0.42	AIO08861.1	Der f 33	Der f 33 allergen (Dermatophagoides farinae)	Tubulin/FtsZ family (AF025)	0.735	0.866
13	A0A0M3KAH2	Tubulin alpha chain	-	-	48,222	5	0.51	AUX14773.1	Der f 33-like	Der f 33-like protein (Dermatophagoides pteronyssinus)	-	0.743	0.891

^1^ Potential allergen and homologous allergens with identity and similarity values (1 is equal to 100%) were calculated using the full-length FASTA searching algorithm by AllergenOnline.org.

**Table 4:** List of known B-cell/T-cell epitopes that were bioinformatically identified in Ani s 1 and Ani s 5 peptides derived from the autoclaved antigen of *A. simplex*.

Peptide ^1^		Epitope ^3^				
Sequence ^1^	Position^1^	IEDB Epitope ID ^3^	Sequence ^3^	Position^3^	B-Cell/T-Cell ^3^	Reference ^3^
**Q7Z1K3 (Ani s 1) ^4^**						
**LFANCCK ^2^**	188‒194	137143	EDAKCERGKLFANCCK^5^	179‒194	B-cell	[48]
		421049	GKLFANCCK	186‒194	T-cell	[49]
**EEELFAR**	137‒143	421391	MGLCCPTKEEELFA	129‒142	B-cell	[49]
		421053	GLCCPTKEEELFA	130‒142	B-cell	[49]
		420891	EELFAREYEGVC	138‒149	T-cell	[49]
		420774	CCPTKEEELFAR	132‒143	T-cell	[49]
**GSGWMMTILGK**	160‒170	137278	RGSGWMMTILGKSCD	159‒173	B-cell	[48]
		421086	GWMMTILGKSCD	162‒173	T-cell	[49]
		421087	GWMMTILGKSCDDQFCPEDA	162‒181	B-cell	[49]
		421233	KMDRGSGWMMTI	156‒167	T-cell	[49]
		421406	MTILGKSCDDQFCPEDA	165‒181	B-cell	[49]
		421613	RGSGWMMTILG	159‒169	B-cell	[49]
**MMAFMGLCCPTK**	125‒136	420792	CPNGYQCKMMAFMG	117‒130	B-cell	[49]
		421053	GLCCPTKEEELFA	130‒142	B-cell	[49]
		421091	GYQCKMMAFMGL	120‒131	T-cell	[49]
		421092	GYQCKMMAFMGLCCPTK	120‒136	B-cell	[49]
		421384	MAFMGLCCPTKE	126‒137	T-cell	[49]
		421391	MGLCCPTKEEELFA	129‒142	B-cell	[49]
**MDRGSGWMMTILGK**	157‒170	137278	RGSGWMMTILGKSCD	159‒173	B-cell	[48]
		421086	GWMMTILGKSCD	162‒173	T-cell	[49]
		421087	GWMMTILGKSCDDQFCPEDA	162‒181	B-cell	[49]
		421233	KMDRGSGWMMTI	156‒167	T-cell	[49]
		421406	MTILGKSCDDQFCPEDA	165‒181	B-cell	[49]
		421613	RGSGWMMTILG	159‒169	B-cell	[49]
**SGICLSFK**	56‒63	420822	DKSGICLSFKYT	54‒65	T-cell	[49]
		421875	WHDDKSGICLSFKY	51‒64	B-cell	[49]
**A1IKL2 (Ani s 5)**						
ADAELSK	105‒111	230144	KKADAELSKIA	103‒113	B-cell	[50]
FETFKK	73‒78	-	-	-	-	-
TDPEIEK	50‒56	-	-	-	-	-
AKEAELAK	82‒89	-	-	-	-	-
KADAELSK	104‒111	230144	KKADAELSKIA	103‒113	B-cell	[50]
AFFELLK	38‒44	-	-	-	-	-
DETKTDPEIEK	46‒56	230147	KTDPEI	49‒54	B-cell	[50]
**DELEKGIGPAVPQ**	140‒152	230136	IQAIYKTLPQSVKDELEKGI	127‒146	B-cell	[50]
**TLPQSVKDELEK**	133‒144	230136	IQAIYKTLPQSVKDELEKGI	127‒146	B-cell	[50]
		230137	IQKAQKIQAIYKTLPQSVKD	121‒140	B-cell	[50]
EAELAKAHEEAVAK	84‒97	-	-	-	-	-
DLDAWVDTLGGDYK	57‒70	230149	LDAWVDTLG	58‒66	B-cell	[50]
		230150	LDAWVDTLGGDYKAKFETFK	58‒77	B-cell	[50]
DLDAWVDTLGGDYKAK	57‒72	230149	LDAWVDTLG	58‒66	B-cell	[50]
		230150	LDAWVDTLGGDYKAKFETFK	58‒77	B-cell	[50]
TDPEIEKDLDAWVDTLGGDYK	50‒70	230149	LDAWVDTLG	58‒66	B-cell	[50]
		230150	LDAWVDTLGGDYKAKFETFK	58‒77	B-cell	[50]

^1^Peptides were identified using LC-MS/MS and retrieved from the Mascot search results; ^2^sequences of unique peptides are bolded; ^3^epitope mapping data were retrieved from the Immune Epitope Database and Analysis Resource (IEDB); ^4^UniProt ID and allergen name; ^5^the epitope sequences found in the peptides are underlined.

**Table 5:** List of in silico predicted epitopes (MHC II, T-cell, and B-cell) in Ani s 2, Ani s 3, and Ani s 4 peptides derived from the autoclaved antigen of *A. simplex*.

Peptide ^1^		Epitope MHC II ^3^		Epitope T-Cell ^3^		Epitope B-Cell ^3^	
Sequence ^1^	Position ^1^	Sequence ^3^	Position ^3^	Sequence ^3^	Position ^3^	Sequence ^3^	Position ^3^
**Q9NJA9 (Ani s 2) ^4^**							
**ANLEAQK^2^**	562‒568	-	-	-	-	-	-
**DLQVALK**	465‒471	-	-	-	-	-	-
SLEEQVK	712‒718	-	-	-	-	-	-
AALAELQK	402‒409	-	-	LAELQKMKQLYEKAVE ^5^	404‒419	-	-
**TALDNAIR**	617‒624	-	-	CKTALDN	615‒621	-	-
ELEDAEGR	829‒836	-	-	-	-	RELEDAEGRA	828‒837
ELHAADER	674‒681	-	-	LDEVTKELHAAD	668‒679	-	-
**ALAELQQVR**	487‒495	-	-	RAQRALAEL	483‒491	-	-
**VALDEESAAR**	271‒280	-	-	-	-	-	-
**HKETQSALR**	760‒768	-	-	-	-	RIRDLEVALDEETRRHKETQSALRKKDRRI	745‒774
LTAALADAEAR	523‒533	LTAALA	523‒528	-	-	-	-
**AQNTIAILER**	361‒370	-	-	-	-	-	-
EEEMEALRK	506‒514	-	-	-	-	-	-
VQLDNLQHVK	222‒231	-	-	-	-	-	-
LHELDLENAR	449‒458	-	-	-	-	-	-
**LLQDDFESER**	43‒52	-	-	-	-	QDDFESERELRNRIERERA	45‒63
**MFVMAQDTADR**	787‒797	-	-	DTADRMLE	793‒800	-	-
**ADQAESSLNLIR**	837‒848	-	-	-	-	-	-
**FEQQTIELSNK**	178‒188	-	-	-	-	-	-
KLHELDLENAR	448‒458	-	-	-	-	-	-
ISALSAELEECK	605‒616	ISALSA	605‒610	-	-	-	-
**YQLAQQLEESR**	232‒242	-	-	-	-	-	-
**QAEADLEEAHVR**	628‒639	AHVRISDLTS	635‒645	-	-	-	-
**EVQMQIDEEHK**	776‒786	-	-	-	-	EVQMQI	776‒781
**LSLANTEITQWK**	287‒298	-	-	-	-	-	-
ADLSVQLIALTDR	63‒75	-	-	-	-	-	-
**DLQVALKESEAAR**	465‒477	-	-	-	-	-	-
KISALSAELEECK	604‒616	ISALSA	605‒610	-	-	-	-
**KQAEADLEEAHVR**	627‒639	-	-	-	-	-	-
**IRDLEVALDEETR**	746‒758	-	-	-	-	RIRDLEVALDEETRRHKETQSALRKKDRRI	745‒774
**ISDLTSINSNLTAIK**	640‒654	AHVRISDLTS	635‒645	ISDLTSI	640‒646	-	-
LQSEVEVLIVDLEK	347‒360	-	-	-	-	-	-
**IKEVQMQIDEEHK**	774‒786	-	-	-	-	EVQMQI	776-781
**QLQQTLDQYALAQR**	590‒603	-	-	LEDTQRQLQQT	584‒594	-	-
ERADLSVQLIALTDR	61‒75	-	-	-	-	-	-
**AVQELHEEQEHSMK**	692‒705	-	-	ANRALADAARAVQELHE	682‒698	-	-
**QLGEAEAMTMQNLQR**	808‒822	-	-	AEAMTMQNLQRVRRYQRELEDA	812‒833	-	-
**SRIDELLVELEAAQR**	384‒398	-	-	IDELLVE	386‒392	-	-
**QAEYEEQIEIMLQK**	323‒336	-	-	-	-	-	-
**VEDLNKHVNDLAQQR**	189‒203	-	-	SNKVEDLNKHVNDLAQ	186‒201	LNKHVN	192-197
**MAQKFEQQTIELSNK**	174‒188	-	-	HVAEKMAQKFE	169‒179	-	-
**QLQQTLDQYALAQRK**	590‒604	-	-	LEDTQRQLQQT	584‒594	-	-
**LQAENSDLLAEIHDQK**	206‒221	-	-	DLLAEIH	212‒218	-	-
**ISDLTSINSNLTAIKNK**	640‒656	AHVRISDLTS	635‒645	ISDLTSI	640‒646	-	-
**ARLQSEVEVLIVDLEK**	345‒360	-	-	-	-	-	-
**LETELSTAQADLDEVTK**	657‒673	LSTAQA	661‒666	LDEVTKELHAAD	668‒679	-	-
**LQHEVIELTATIDQLQK**	150‒166	IELTAT	155‒160	EVIELTATIDQLQK	153‒166	-	-
LLEESQLENEDAMNVLR	103‒119	-	-	EDAMN	112‒116	-	-
YQAEIAELEMTVDNLNR	545‒561	-	-	EMTVDNLN	553‒560	-	-
QLQVQIQEAEAAALLGGKR	719‒737	-	-	-	-	-	-
**FEQQTIELSNKVEDLNK**	178‒194	-	-	SNKVEDLNKHVNDLAQ	186‒201	-	-
**VEAEHKLSLANTEITQWK**	281‒298	-	-	-	-	-	-
**NKLETELSTAQADLDEVTK**	655‒673	LSTAQA	661‒666	LDEVTKELHAAD	668‒679	-	-
**SMQFEIDRLTAALADAEAR**	515‒533	LTAALA	523‒528	-	-	-	-
**QRLQAENSDLLAEIHDQK**	204‒221	-	-	DLLAEIH	212‒218	-	-
**SQMQAQLHQVQLELDSVR**	253‒270	LHQVQL	259‒264	-	-	-	-
SKFDAEVALHHEEVEDLR	299‒316	-	-	EVEDLRKKMMQ	311‒321	-	-
**SQMQAQLHQVQLELDSVR**	253‒270	LHQVQL	259‒264	-	-	-	-
LLEESQLENEDAMNVLRK	103‒120	-	-	EDAMN	112‒116	-	-
KLLEESQLENEDAMNVLR	102‒119	-	-	EDAMN	112‒116	-	-
**QRLQHEVIELTATIDQLQK**	148‒166	-	-	EVIELTATIDQLQK	153‒166	-	-
QAEYEEQIEIMLQKVSQLEK	323‒342	-	-	-	-	-	-
**MKAEIAR**	534‒540	-	-	-	-	-	-
**MKQLYEK**	410‒416	-	-	-	-	-	-
**EVELSKLR**	94‒101	-	-	-	-	ESNRKREVELS	88‒98
**DLEVALDEETRR**	748‒759	-	-	-	-	RIRDLEVALDEETRRHKETQSALRKKDRRI	745‒774
**LQDAECATDSQIESNR**	76‒91	-	-	-	-	ESNRKREVELS	88‒98
**KQSEQIIQLQANLEDTQR**	572‒589	IIQLQA	577‒582	LEDTQRQLQQT	584‒594	-	-
**SSTADMGALTSMSVADLGSLTR**	15‒36	LTSMSVA	23‒29	MGALTSMSVADLGSLTRL	20‒37	-	-
**ISALSAELEECKTALDNAIR**	605‒624	ISALSA	605‒610	CKTALDN	615‒621	-	-
VSQLEK	337‒342	-	-	-	-	-	-
**LNIQKR**	802‒807	-	-	-	-	-	-
**KSLEEQVK**	711‒718	-	-	DALRKSLEEQV	707‒717	-	-
KYQAEIAELEMTVDNLNR	544‒561	-	-	EMTVDNLN	553‒560	-	-
Q9NAS5 (Ani s 3)							
**AEFAER**	239‒244	-	-	-	-	-	-
**ADAAEEK**	22‒28	-	-	DAAEEKVRQMTDKLERIEEELRDTQKKMMQTENDLDKAQEDL	23‒64	-	-
**KVMENR**	128‒133	-	-	-	-	RKVMENRSFQDEE	127‒139
**IEEELR**	39‒44	-	-	-	-	-	-
**LKEAETR**	232‒238	-	-	-	-	-	-
SFQDEER	134‒140	-	-	-	-	RKVMENRSFQDEE	127‒139
SLEVSEEK	206‒213	-	-	-	-	-	-
ANTVESQLK	141‒149	-	-	-	-	-	-
**QMTDKLER**	31‒38	-	-	-	-	TDKLERIEEELRDT	33‒46
**IEKDNALDR**	13‒21	-	-	-	-	-	-
**LEDELVHEK**	256‒264	-	-	EFAERSVQKLQKEVDRLEDELVH	240‒262	DRLEDELV	254‒261
IVELEEELR	190‒198	-	-	KIVELEE	189‒195	LEEEL	193‒197
**AEFAERSVQK**	239‒248	-	-	EFAERSVQKLQKEVDRLEDELVH	240‒262	-	-
EDSYEEQIR	218‒226	-	-	EQIRTVS	223‒229	LQREDSYEEQIR	215‒226
**MMQTENDLDK**	50‒59	-	-	-	-	-	-
**IEEELRDTQK**	39‒48	-	-	-	-	TDKLERIEEELRDT	33‒46
EAQMLAEEADR	150‒160	-	-	-	-	-	-
MTLLEEELER	92‒101	-	-	-	-	-	-
SLEVSEEKALQR	206‒217	-	-	-	-	-	-
EAQMLAEEADRK	150‒161	-	-	-	-	-	-
**VQEAEAEVAALNR**	78‒90	-	-	-	-	-	-
**DNALDRADAAEEK**	16‒28	-	-	DAAEEKVRQMTDKLERIEEELRDTQKKMMQTENDLDKAQEDL	23‒64	-	-
RMTLLEEELER	91‒101	-	-	-	-	-	-
LEEATHTADESER	113‒125	-	-	LEEATHTADESERVRKVME	113‒131	-	-
**KVQEAEAEVAALNR**	77‒90	-	-	-	-	-	-
**VQEAEAEVAALNRR**	78‒91	-	-	-	-	-	-
**EVDRLEDELVHEK**	252‒264	-	-	EFAERSVQKLQKEVDRLEDELVH	240‒262	DRLEDELV	254‒261
ALQREDSYEEQIR	214‒226	-	-	EQIRTVS	223‒229	LQREDSYEEQIR	215‒226
**AQEDLSTANSNLEEK**	60‒74	-	-	-	-	-	-
**SISEELDQTFQELSGY**	269-284	-	-	-	-	-	-
**YKSISEELDQTFQELSGY**	267‒284	-	-	-	-	-	-
**ANTVESQLKEAQMLAEEADR**	141‒160	-	-	-	-	-	-
**KMMQTENDLDK**	49‒59	-	-	-	-	-	-
**MMQTENDLDKAQEDLSTANSNLEEK**	50‒74	-	-	DAAEEKVRQMTDKLERIEEELRDTQKKMMQTENDLDKAQEDL	23‒64	-	-
Q14QT4 (Ani s 4)							
**KQVVAGDK**	69‒76	-	-	-	-	-	-
**KWENFEEVK**	100‒108	-	-	WQKKWENFEEVKVLKCDH	97‒114	-	-
**ISAMINDGKPHELVK**	49‒63	-	-	ELAGKSIAKISAMI	40‒53	-	-
		-	-	GKPHELVKVVS	56‒66	-	-
**ELAGKSIAK**	40‒48	-	-	ELAGKSIAKISAMI	40‒53	-	-
**WENFEEVK**	101‒108	-	-	WQKKWENFEEVKVLKCDH	97‒114	-	-

^1^ Peptides were identified using LC-MS/MS and retrieved from the Mascot search results; ^2^ sequences of unique peptides are bolded; ^3^ major histocompatibility complex class II (MHC II) molecules, T-cell, and B-cell epitopes were predicted using DNA STAR Protean 3D software; ^4^ UniProt ID and allergen name; ^5^ the epitope sequences found in the peptides are underlined.

## Supplementary Materials

The following are available online at https://www.mdpi.com/2218-273X/10/7/1066/s1, **File S1:** The list of all proteins identified using LC-MS/MS, and results of the functional analysis performed by OmicsBox software; **File S2:** The structural alignments of the following potential allergens and their homologous allergens (native structures): A0A0M3KCE6 (97% of residues modeled at > 90% confidence, model dimensions in angstroms (Å): X:27.759 Y:173.490 Z:45.247) vs. Asc l 3 (**a**), A0A0M3KA05 (81% of residues modeled at > 90% confidence, model dimensions in Å: X:75.957 Y:58.757 Z:35.306) vs. Ani s 8 (**b**), A0A158PP35 (89% of residues modeled at > 90% confidence, model dimensions in Å: X:125.866 Y:222.697 Z:221.902) vs. Ani s 2 (**c**), A0A0M3J4T5 (67% of residues modeled at > 90% confidence, model dimensions in Å: X:47.481 Y:73.842 Z:41.442) vs. Ani s 2 (**d**), A0A0M3J693 (97% of residues modeled at > 90% confidence, model dimensions in Å: X:46.182 Y:84.389 Z:117.031) vs. Asc l 3 (**e**), A0A0M3K9V2 (95% of residues modeled at > 90% confidence, model dimensions in Å: X:65.744 Y:74.745 Z:102.212) vs. Tyr p 28 (**f**), A0A0M3KIW1 (97% of residues modeled at > 90% confidence, model dimensions in Å: X:52.953 Y:70.661 Z:62.596) vs. Tyr p 28 (**g**), A0A0M3J6G4 (92% of residues modeled at > 90% confidence, model dimensions in Å: X:39.808 Y:40.355 Z:35.353) vs. Ole e 15 (**h**), A0A0M3JU57 (91% of residues modeled at > 90% confidence, model dimensions in Å: X:43.290 Y:44.288 Z:50.719) vs. Ani s troponin C (**i**), A0A0M3K5H6 (89% of residues modeled at > 90% confidence, model dimensions in Å: X:120.529 Y:61.462 Z:88.699) vs. Aed a 8 (**j**), A0A0M3J8H0 (53% of residues modeled at > 90% confidence, model dimensions in Å: X:49.537 Y:38.719 Z:27.443) vs. Ani s 9 (**k**), A0A0M3K821 (90% of residues modeled at > 90% confidence, model dimensions in Å: X:62.766 Y:54.503 Z:55.905) vs. Der f 33 (**l**), A0A0M3KAH2 (91% of residues modeled at > 90% confidence, model dimensions in Å: X:71.490 Y:51.790 Z:60.479) vs. Der f 33-like (**m**). Models of unknown 3D structures were performed using the Phyre2 server. Model dimensions, coverage, and confidence of structure prediction of homologous allergens for potential allergens are reported in Figure 5 and Figure 6. PyMOL software was used to perform alignment and visualize all structures. Potential allergen structure is presented in cartoon view mode and highlighted in white, while the homologous allergen structure is presented in the ribbon mode view and highlighted in red.
