# Supplementary material for: Proteomic and Bioinformatic Investigations of Heat-Treated Anisakis simplex Third-Stage Larvae
Source: Biomolecules. 2020 Jul 16;10(7):1066. doi: 10.3390/biom10071066 (PMC7407331; doi:10.3390/biom10071066)
Supplement: Supplementary file 1 [file biomolecules-10-01066-s001.zip › Supplemental File S2 2020-05-24.pdf]

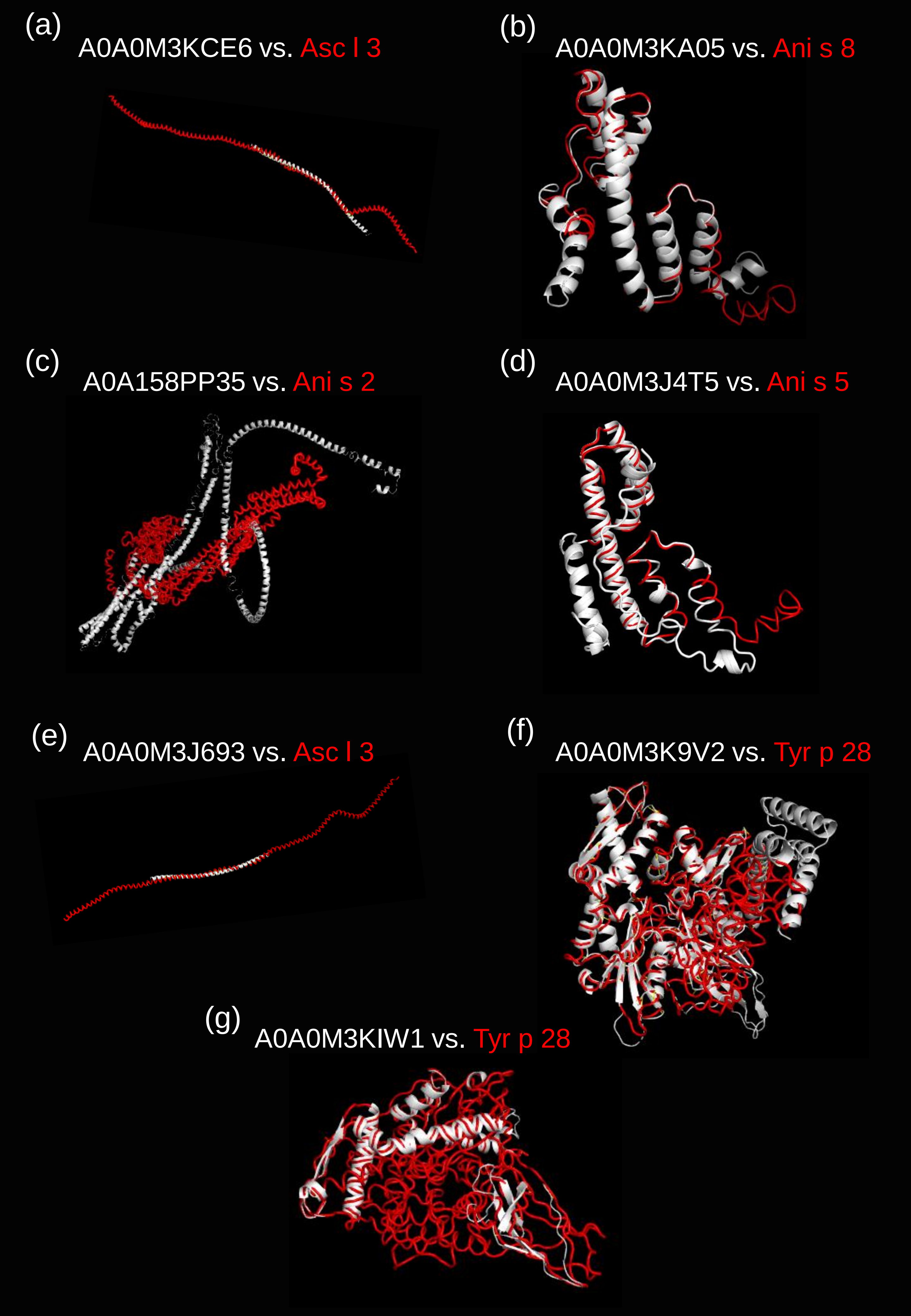
(a)
A0A0M3KCE6 vs. Asc l 3
(b)
A0A0M3KA05 vs. Ani s 8
(c)
A0A158PP35 vs. Ani s 2
(d)
A0A0M3J4T5 vs. Ani s 5
(e)
A0A0M3J693 vs. Asc l 3
(f)
A0A0M3K9V2 vs. Tyr p 28
(g)
A0A0M3KIW1 vs. Tyr p 28

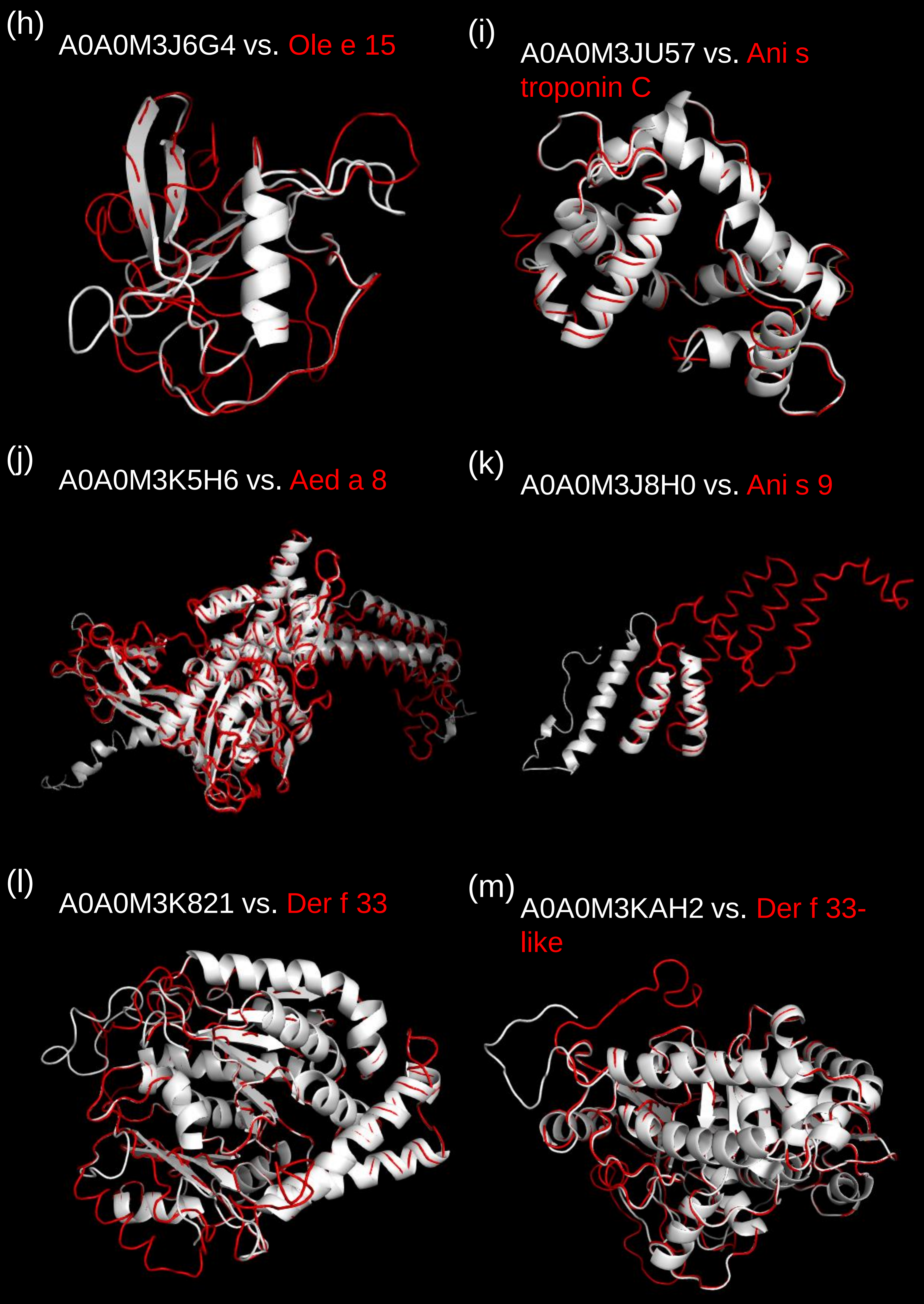
(h)
A0A0M3J6G4 vs. Ole e 15
(i)
A0A0M3JU57 vs. Ani s troponin C
(j)
A0A0M3K5H6 vs. Aed a 8
(k)
A0A0M3J8H0 vs. Ani s 9
(l)
A0A0M3K821 vs. Der f 33
(m)
A0A0M3KAH2 vs. Der f 33-like
